# Development of actionable quality indicators and an implementation toolkit for perioperative opioid stewardship in colorectal cancer in the UK Yorkshire and Humber region: a modified RAND consensus study

**DOI:** 10.1136/bmjopen-2024-092675

**Published:** 2025-09-30

**Authors:** Sarah Alderson, Caroline Thomas, Hannah Rossington, Emily Connearn, Simon Howell

**Affiliations:** 1 Leeds Institute of Health Sciences, University of Leeds, Leeds, West Yorkshire, UK; 2 Leeds Teaching Hospitals NHS Trust, Leeds, West Yorkshire, UK; 3 Leeds Institute of Data Analytics, University of Leeds, Leeds, West Yorkshire, UK; 4 Leeds Institute of Medical Research, University of Leeds Faculty of Medicine and Health, Leeds, West Yorkshire, UK; 5 Leeds Institute of Biomedical and Clinical Sciences, University of Leeds, Leeds, West Yorkshire, UK

**Keywords:** Implementation Science, Quality Improvement, Quality in health care

## Abstract

**Abstract:**

**Objectives:**

There are global concerns about the rise in opioid prescribing. Patients undergoing potentially curative surgery for colorectal cancer (CRC) are at high risk of adverse outcomes from opioid-related complications, including delayed discharge and adjuvant chemotherapy, long-term opioid use and reduced cancer-free survival. We aimed to develop a set of actionable quality indicators for opioid stewardship for patients undergoing CRC surgery, and an implementation toolkit to support professional behaviour change to improve appropriateness of perioperative opioid prescribing.

**Design:**

A five-round modified RAND consensus process was conducted in 2021–2024.

**Setting:**

14 secondary care trusts across the UK Yorkshire and Humber region.

**Participants:**

Consultant anaesthetists and national perioperative opioid stewardship experts (expert panel) and patient and public panel.

**Interventions:**

Potential indicators were identified from a literature review, guideline search and expert panel. All potential indicators were rated on relevance and actionability (online survey, expert panel) and importance to patient care (online meeting, patient panel). A hybrid consensus meeting involving a patient representative and the expert panel discussed and rerated the indicators. An online expert survey identified potential barriers to implementation. An actionable toolkit was developed using implementation strategies and supporting resources developed where appropriate.

**Results:**

73 potential indicators were identified. All indicators remained in the process through the online survey and patient panel. After the final meeting, four indicators remained: (1) hospital trust presence of an opioid stewardship protocol; (2) inpatient functional post-operative pain assessments; (3) patient education and discharge leaflet; and (4) senior clinician review of ‘strong’ opioids on discharge (British National Formulary definition). The number of barriers identified ranged from 8 to 22 per indicator. 49 different implementation strategies were identified for the toolkit (range 32–45 per indicator).

**Conclusions:**

We identified four actionable quality indicators and developed an implementation toolkit that represents consensus in defining quality of care in opioid stewardship for CRC surgery.

**STRENGTHS AND LIMITATIONS OF THIS STUDY**

- The strength of this study on opioid stewardship in colorectal cancer surgery is the following of the systematic process of a modified RAND consensus process, which includes both evidence and expert opinions, and involved patients throughout.
- Not all panel members completed every stage of the consensus process, with only 8 of the 10 expert panel members attending the consensus meeting and barrier identification for the toolkit.
- Earlier patient involvement in the generation of the initial list of indicators may have identified other potential indicators.
- Involving other members of the healthcare team, such as surgeons and specialist colorectal nurses, may have led to different results and support with future implementation into routine care.

## Introduction

 In the UK, rates of opioid use are known to be increasing, with a corresponding increase in reports of related harms, such as mortality and morbidity.^1^ The USA, Germany and Canada have reduced their opioid prescribing, leaving the UK with the highest consumption rate of prescription opioids for pain management per capita in the world.^2^ Inappropriate prescribing following surgery is increasingly recognised as contributing to the rise in opioid prescribing. Opioids are effective analgesics for managing acute pain in the perioperative period^3^ and have been used in longer and higher doses following the publication of guidelines on post-operative pain management.^4^ However, opioids also have significant adverse effects, including sedation, constipation, nausea and confusion, which slow recovery from surgery and contribute to long-term opioid use.^5^,^7^ Patients undergoing potentially curative surgery for colorectal cancer are at a particularly high risk of adverse outcomes from opioid-related complications leading to delayed or omitted adjuvant chemotherapy,^8 9^ increased rates of local recurrence and reduced cancer-specific survival.^10 11^

A requirement for an effective opioid stewardship programme in perioperative care is the potential to determine the appropriateness of opioid use. Quality indicators measure the quality of the process, performance or outcome of healthcare delivery.^12^ Features of good quality indicators include their relevance, feasibility and reliability. Indicators should be easy to understand and agreed by key interested parties such as the multidisciplinary team (MDT) for colorectal cancer, managers, commissioners and patients, which are achievable by changing behaviours and measurable with high validity.^13^ They should have a good evidence base and a high correlation with quality of care.

Quality indicators are frequently used to measure the variability in the quality of care and identify where areas of improvement and further resources may be needed through feedback of achievement to healthcare teams.^14^ Tailored multifaceted interventions are designed to improve quality of care by addressing barriers in the different levels of the specific healthcare system context (including organisation, team, professionals and patients).^15^ Systematic development of a tailored multi-faceted intervention involves identifying local barriers and then developing interventions that target the barriers identified. However, healthcare professionals may not have the knowledge, time and skills to develop and implement improvement strategies.^16 17^ Healthcare professionals can be supported by the provision of a list of potential barriers and their associated improvement strategies (an implementation toolkit).^18 19^

Improving opioid stewardship during the perioperative period for patients with colorectal cancer has the potential to improve recovery, lead to faster discharge and improved outcomes and, most importantly, prevent patient harm. The Yorkshire Cancer Research (YCR) Bowel Cancer Improvement Programme (BCIP) aims to improve survival from colorectal cancer across the Yorkshire and Humber region.^20^ Outcomes from colorectal cancer in the region are comparable to those for the rest of the UK but not as good as elsewhere in Europe.^21^ The YCR BCIP programme works to both support the practice of individual clinicians and optimise local clinical systems.

This study aimed to develop and agree through consensus a set of actionable quality indicators and a multilevel implementation toolkit, which can be used to assess and improve regional opioid stewardship in the management of patients with colorectal cancer undergoing surgery.

## Methods

**Figure 1:**
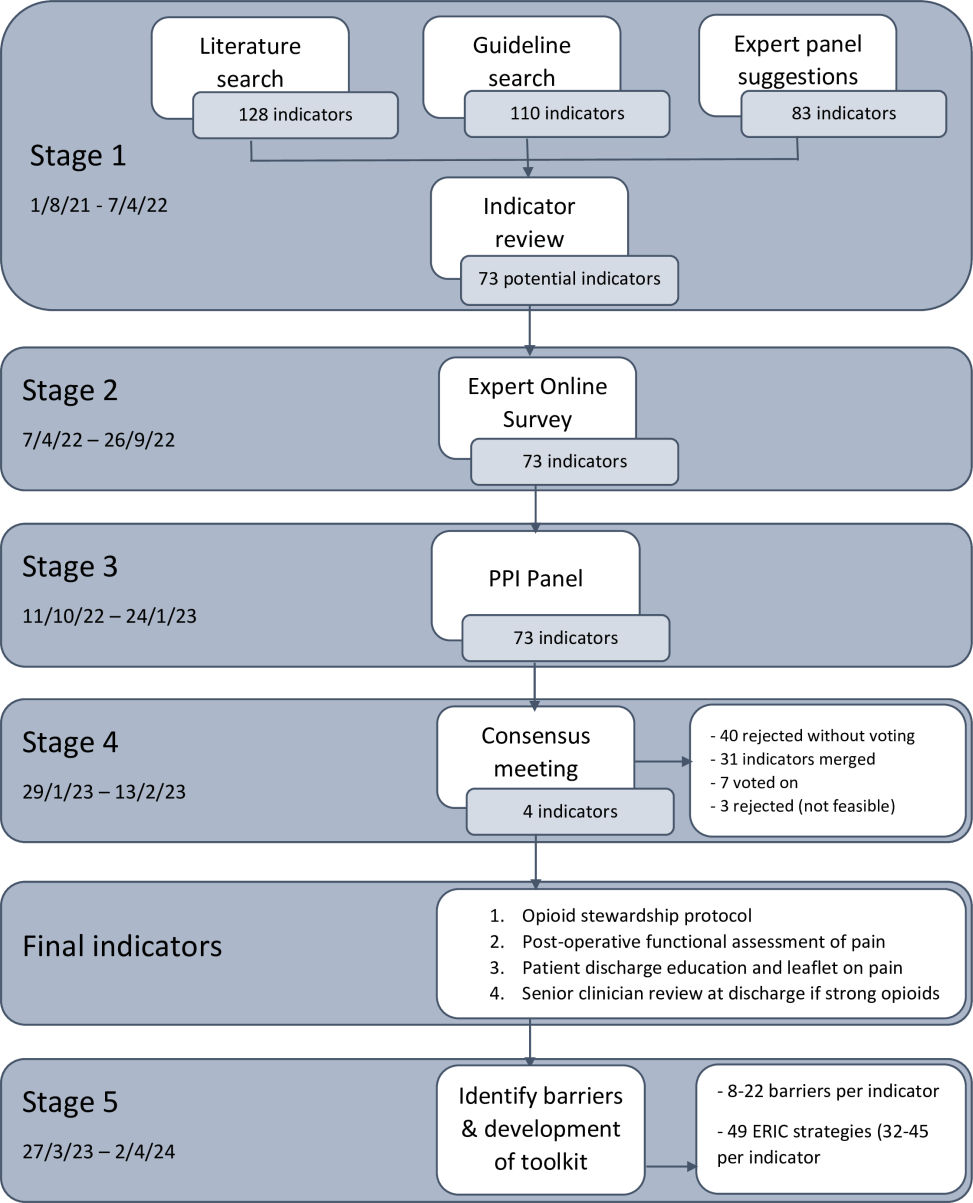
Overview of modified RAND method and study results. ERIC, Expert Recommendations for Implementing Change. PPI, patient and public involvement.

We followed best practice in quality indicator development^13^ and have attached the ACcurate COnsensus Reporting Document (ACCORD) checklist (online supplemental material 1). We used a modified RAND consensus process,^13 22 23^ consisting of five stages, involving patients and experts, to develop quality indicators and an action toolkit for appropriate opioid use in patients undergoing surgery for colorectal cancer. The research team was led by the lead implementation science (SA) and anaesthetic (SH) workstreams and the programme manager (HR) for BCIP, and a consultant anaesthetist with an interest in perioperative analgesia (CT). The plans for the study were discussed at a regional opioid management update event but not prospectively registered. The research team did not take part in the ranking exercises and were able to view individual scores; however, these were not visible to other participants. Figure 1 shows a summary of the modified RAND process.

### Patient and public involvement

The acceptability and utility of quality indicators depend on stakeholders’ perception of indicator relevance and value.^14^ Therefore, including the voices of patients and the public is important. BCIP jointly funds with CORECT-R programme an active Patient and Public Group (PPG), Bowel Cancer Intelligence UK (BCI-UK), with a patient representative within the core research team. The patient representative attended monthly BCIP meetings where study design and outcomes were discussed. The full PPG were involved in Stage 3 and the patient representative additionally in Stage 4 of this study.

### Stage 1: identification of potential indicators

We conducted a rapid systematic literature search in Medline to identify existing indicators on appropriate opioid use in colorectal cancer surgery following Cochrane Rapid Review methods.^24^ The search included all articles available in Medline up to April 2021. Search terms included terms and truncations for quality indicators, opioids, surgery (with potential limitation to colorectal cancer surgery) and development. The search was limited to English-language primary studies of adults and reviews and supplemented with manual reference searches and consultation of the National Quality Measures Clearinghouse.^25^ From 588 abstracts, three members of the literature review team used the Rayyan platform for title and abstract screening, applying inclusion criteria, and reviewed 47 full-text publications that contained extractable indicators. The full text of relevant articles was reviewed by the full project team for potential quality indicators. Using a bespoke data extraction tool, we mapped indicators to stages of the perioperative pathway, removed duplicates and derived 24 discrete quality indicators. We then categorised these as structure, process or outcome measures, and grouped them into five thematic areas: patient education, clinician education, pre-operative optimisation, procedure-specific prescribing and deprescribing, and opioid-related adverse drug events. We have published the full results of our rapid review.^24^

We invited panellists based on their (inter)national involvement in guideline development regarding opioid use in (colorectal) surgery for their expertise (n=2) and key local leaders for quality improvement in colorectal cancer care (MDT lead anaesthetists and surgeons) for their real-world knowledge of barriers to, and implementation of, potential quality indicators (n=31) from 14 National Health Service trusts in the Yorkshire and Humber region (population 5.4 million). All potential panellists were invited by email and written consent was obtained from those who agreed to form the expert panel. Panellists were instructed to individually suggest by email potential quality indicators representing appropriate perioperative opioid use in colorectal cancer surgery, based on their knowledge and expertise in this area.

In addition, we made an expert-based selection of national and international guidelines regarding opioid use in patients undergoing cancer surgery, from which we extracted all potential indicators. Finally, we collated a list of all potential indicators. Indicators were rephrased and merged if appropriate and duplicate indicators removed.

### Stage 2: expert online survey

We used online surveys^26^ to deliver the list of potential quality indicators by email to all expert panellists. We asked the expert panel members to score each indicator individually on a 9-point Likert scale (1=totally disagree, 9=totally agree), against two criteria: (1) relevance—the impact of the indicator on colorectal cancer care or on cost-effectiveness and (2) actionability—the extent to which an indicator recommends improvement in usual clinical practice and is under the recipient’s control (online supplemental material 2). Indicators that had a median score between 4 and 9 on both relevance and actionability were kept as potential quality indicators. Indicators that had a median score of 1–3 on either relevance or actionability were retained to discuss in Stage 3 with our patient and public involvement panel.

### Stage 3: patient and public group (PPG) online panel

All members of the BCIP PPG (n=10) were invited to take part in the online PPG panel with information on the study and consent form attached to an email. The PPG members who consented to join the panel were sent the list of all potential quality indicators identified in Stage 1 along with the median and range of scores from the Stage 2 expert survey prior to the meeting with explanations of terminology where needed.

During an online meeting, each potential indicator was discussed (online supplemental material 3), and the PPG panel asked to rate the indicator individually on importance to patient care on a Likert scale of 1 (not important) to 9 (very important). Indicators not reaching inclusion from the expert panel were discussed and rated. Those that were rated 4–9 on importance to patient care by the PPG panel were retained for future discussions with the BCIP core research team for further quality improvement action but not as a potential quality indicator. All indicators with a median score between 4 and 9 from both the PPG panel and expert panel were taken forward to the next stage.

### Stage 4: in-person consensus meeting

We presented the results from Stages 2 and 3 in a face-to-face meeting in Leeds, West Yorkshire, to the expert panel and a nominated PPG representative from the PPG panel following accepted methodology for RAND consensus processes.^23^ Panel members were sent the list of potential indicators and expert and PPG panel scores from Stages 2 and 3 in advance of the meeting (online supplemental material 4). The indicators were reviewed, rephrased, merged and scored for the second time based on four criteria: (1) relevance; (2) actionability; (3) feasibility of data collection, including the ability to measure using routinely collected electronic data; and (4) validity, reflecting whether the indicator measures quality of opioid use in clinical practice (ie, face and content validity). A blinded survey tool (AHASlides^27^) supported independent rating.

After the second rating, all indicators with median scores in the top (7 to 9) tertile on relevance, actionability, feasibility and validity without disagreement were retained. Agreement was defined as 60% or more scores in the top tertile.^28^ The research team described each indicator in detail, including definitions, inclusion and exclusion criteria and supportive evidence for good practice. The final indicators were sent to all expert panel members for approval.

### Stage 5: development of an implementation toolkit

The research team discussed determinants of practice that might act as barriers to healthcare professionals improving performance for each indicator using the Consolidated Framework of Implementation Research (CFIR) constructs.^18 19^ All consensus panel members were asked to rate whether CFIR constructs were barriers to achieving each indicator in an online survey. Barriers were kept if 33% or more of participants rated the construct as a potential barrier to achievement. We developed a list of improvement strategies to overcome these specific barriers using the CFIR Expert Recommendations for Implementing Change (ERIC).^29^ Where available, resources to support the improvement strategies were collated for each strategy (eg, a suggested template for an opioid stewardship protocol, patient leaflets). The BCIP implementation toolkit comprising of the list of barriers, their associated improvement strategies and resources was sent to all panel members for review and additional suggestions.

## Results

### Stage 1: identification of potential indicators

The methods and results of the literature search have been described elsewhere.^24^ The search identified 558 papers, of which 83 papers had full-text review. 30 papers met inclusion criteria and had their references reviewed, leading to review of a further 35 full-text papers, of which 17 additional papers were included. In total, 128 potential quality indicators were extracted from the 47 included papers. 12 relevant guidelines were identified with 110 potential indicators extracted. The expert panel additionally suggested 83 potential indicators. All 420 potential indicators from the three sources were reviewed for duplication by the research team, grouped and rephrased where necessary to give 73 potential indicators for the online survey. The full list of 73 indicators and the scores at each following stage is given in online supplemental table 1.

### Stage 2: expert online survey

10 participants formed the expert panel and responded to the online survey. These included nine colorectal cancer MDT anaesthetic leads from eight trusts within the BCIP region and one national expert on perioperative opioid use. All panel members ranked all 73 potential indicators on relevance and actionability. There was a high level of intra-panel agreement with <2% of indicators showing disagreement on relevance and no indicators showing disagreement on actionability. Disagreement was defined as 30% or more scores in both the top (7–9) and bottom (1–3) tertiles. Four indicators were ranked as not actionable with median scores <5.

### Stage 3: patient and public group (PPG) panel

Six PPG members attended the online panel meeting and discussed and rated 73 indicators on importance to patient care. A further two members completed the rating through an online questionnaire. All indicators were rated as important to patients and taken through to the next stage. Notes were taken on discussions to provide context to scores. There was no disagreement within the PPG panel. All indicators were ranked according to their overall scores from stages 2 and 3.

### Stage 4: consensus meeting

A hybrid meeting was held and attended by six members of the expert panel and one PPG representative in-person, and two members of the expert panel joined online. 69 indicators were discussed individually during the meeting, with the four indicators rated as ‘not actionable’ being excluded at this stage. 40 indicators were rejected after discussion. It was agreed that 31 indicators were related and, after re-phrasing, were merged into five existing indicators. A further two indicators were rephrased. The five merged indicators and two rephrased indicators were taken forward for scoring. Four indicators had a median score of 7–9 on all four of relevance, actionability, feasibility of data collection and validity, with agreement. Two indicators were rejected for median scores <7 and one indicator was rejected for not reaching agreement (<60% scoring 7–9). Notes were taken on discussions to provide context to scores.

The final agreed set of four indicators includes one structural indicator and three process indicators:

1. Presence of an opioid stewardship protocol.
2. Documented regular post-operative functional assessment of pain.
3. At discharge, the patient has a discussion regarding their discharge medication and given education and leaflet.
4. Senior clinician review before discharge if discharged with new strong opioids (British National Formulary definition).

All four indicators had theoretical optimum targets of 100%, meaning that it may not be realistic to achieve 100%. However, this should be the aim within the YCR BCIP trusts. All 10 expert panel members and the PPG panel agreed on the final set of indicators and targets (table 1).

**Table 1:** A final list of actionable quality indicators and quality metric for opioid stewardship following surgery for colorectal cancer

	Quality indicator	Indicator type	Definition	Numerator	Denominator	Target value
1	Presence of a Trust perioperative opioid stewardship protocol for colorectal cancer surgery	Structural	Presence of an opioid stewardship protocol to reduce perioperative opioid use in colorectal cancer surgery, covering the patient journey through pre-operative assessment, operative, recovery and post-operative inpatient care, discharge and follow-up of patients, including use of multimodal analgesia, adjuncts such as Non-Steroidal Anti-Inflammatories (NSAIDs) and medications that may limit pain experience such as antiemetics for prescribing clinicians and care staff.	n/a	n/a	100%
2	Perform post-operative functional assessment of pain	Process	Percentage of patients with documented regular post-operative functional assessment of pain which includes ability to cough and deep breathe, and sedation score.	Number of patients with documented post-operative functional assessment of pain at minimum twice weekly.	All patients who have undergone surgery for colorectal cancer, excluding those who are intubated (eg, on Intensive Care Units), or unable to comply with instructions (severe dementia or learning disabilities), or not having opioid medication.	100%
3	Patients given pain education and leaflet on discharge	Process	Percentage of patients who have had a discussion regarding their discharge medication and given education and leaflet on safe administration, storage, weaning, disposal of unused opioids, avoidance of opioid diversion and point of contact if ongoing pain issues.	Number of patients with documented discussion and given pain education leaflet regarding opioid management before discharge.	All patients who have been discharged following surgery for colorectal cancer, excluding those who are unable to read or understand and have no carer to support reading or not discharged with opioid medication.	100%
4	Perform senior review if discharged on strong opioids	Process	Percentage of patients discharged on strong opioids (British National Formulary definition) with a senior clinician review before discharge.	Number of patients with a senior clinician review of need for opioid medication at discharge documented before discharge.	All patients who have been discharged following surgery for colorectal cancer, excluding those who are taking the same dose of strong opioid pre-operatively, for a condition other than colorectal cancer.	100%

### Stage 5: implementation toolkit

Eight members of the panel completed the survey to confirm the wording of the indicators and the potential barriers to achievement. Identified barriers for each indicator ranged from 8 to 22 barriers (online supplemental table 2). A total of 49 ERIC strategies were identified for the toolkit (range 32–45 per indicator, online supplemental table 3). The implementation toolkit gives details for each indicator including definition, numerator, denominator, quality metric, evidence for the indicator, best practice guidance, possible barriers to achievement and suggested implementation strategies to overcome these. Supportive resources developed by the research team (online supplemental table 3) were included in the toolkit where identified. These include a draft opioid stewardship protocol for individual trusts to modify for local requirements and a draft patient educational leaflet on managing pain and opioid medication on discharge. This has been co-produced by the research team and the PPG panel.

## Discussion

We systematically developed four actionable quality indicators and an implementation toolkit to support opioid stewardship following surgery for colorectal cancer. The toolkit contains potential barriers to achievement and specifies implementation strategies to overcome each barrier.

We involved experts early in the process, leading to a high commitment from the panel throughout the process. The continued focus on actionability of the quality indicators, combined with the panel’s involvement in identifying barriers to implementation, should promote the implementation of the quality indicators into routine clinical practice. The quality indicators are suitable for inclusion in performance feedback to healthcare teams to encourage professional behaviour change, and the effectiveness of the indicators and toolkit will be assessed in future studies.

Our quality indicators are similar to some of the guidance within Levy *et al*’s consensus statement on the prevention of opioid-related harm in adult surgical patients.^6^ The recommended strategies to prevent opioid-related harm should continue to be priorities of healthcare teams. Our suggested quality indicators operationalise the suggestions that are currently actionable and feasible for measurement and are prioritised by patients, local and national leads. The indicators that reached consensus for importance and actionability, but not feasibility, will require further work to enable extraction from routine data within electronic health record (EHR) systems to support local quality improvement. For example, discussions with patients regarding realistic expectations of pain and pain management (indicator 1, online supplemental table 1) were rejected as not being feasible to evaluate from current routinely recorded data; personalised discussions with an individual patient cannot be adequately represented by a ‘tick-box’. In other studies, policy interventions introduced with best intentions, such as screening of patients for depression in chronic disease reviews, have shown variable quality of those discussions, and professionals and patients may subvert the process recommended by national guidance, leading to unintended consequences.^30 31^ Process indicators on adherence to an opioid stewardship protocol were rejected with the structural indicator, ‘Presence of an opioid stewardship protocol’ being described by the expert participants as a ‘major step forward in opioid stewardship’ in itself. Currently, only 1 of the 14 trusts represented at the panel meeting has such a protocol. Our toolkit addresses this by providing a modifiable protocol that can be tailored to the needs and resources of individual trusts.

Several indicators were ranked highly in importance to patient care but were not ranked as actionable to implement. These included indicators that required either extensive data extraction from EHR systems that could not be feasibly extracted automatically given current EHR limitations would not be achievable in a resource-limited healthcare system despite all opioids having risks of adverse effects (indicator 53—senior review of need for discharge opioid medication) or would require time-consuming extraction by hand or from primary care EHR (indicators 60—quarterly prescribing reports and 66—prescriptions of opioids after discharge). Process indicators were preferred over patient outcome indicators as they are more feasible to measure and for the hospital teams to action. Outcome indicators such as indicator 63 (patient still taking opioids at 90 days to be reviewed) were not feasible to measure within existing EHRs that do not link primary and secondary care data or the time frame to outcome was considered too long. Improvements to EHRs, including increased search functionality and compatibility of searches across EHR systems, are needed to ensure that quality indicators deemed important for patient care are feasible to implement. Other actionability issues included resource-intensive indicators that implementing would lead to delays in surgery and impact on other areas of the healthcare system (indicators 7 (referral for psychological support for patients with complex pain needs) and 11 (weaning pre-operative opioids), online supplemental table 1). Consideration needs to be given by commissioners to ensure best practice care is adequately resourced and available to patients to improve patient outcomes.

The strength of this study is the following of the systematic process of a modified RAND consensus process, which includes both evidence and expert opinions, and has previously been used to successfully develop quality indicators in other areas.^14 22 32^ We modified the approach described by RAND Corporation^23^ in two ways: first, to ask the expert panel for suggestions of potential indicators without showing them the current literature and guidelines in this area, which allowed them to identify indicators applicable to routine practice; and second, to involve patients throughout the project to ensure that the indicators developed are important to patients.

There are three main limitations to our study. First, not all panel members completed every stage of the process, with only 8 of the 10 expert panel members attending the consensus meeting and barrier identification for the toolkit. Despite this, we had involvement from key opinion leaders from participating trusts across the Yorkshire and Humber region, suggesting that this is an important topic to both patients and practitioners and the final indicator set is suitable for implementation across the region and more widely due to the inclusion of international literature and guidance. In addition, national experts were involved since the initial stage of development and international standard processes were followed throughout. The relevance to other healthcare systems is uncertain, as is relevance to other abdominal operations beyond surgery for colorectal cancer.

Second, our patient panel was not involved in the initial stage of indicator identification. Earlier involvement in the generation of the initial list of indicators may have identified different potential indicators. The patient panel was able to see the expert scores when undertaking the ranking of indicators which may have influenced their response. Despite this, several indicators were scored differently, suggesting that the influence was minimal. For example, an area of disagreement between the expert panel and the PPG panel was the use of patient educational leaflets in the pre-operative period. The expert panel survey ranked pre-operative patient education leaflets on pain management as having high relevance with a median score of 8, whereas the patient panel gave this a median score of just 5. Discussions in the patient panel described how ‘too many leaflets are given pre-operatively which can lead to overwhelm of information’. However, the final included indicator of a patient educational leaflet on pain management following discharge was thought to be more helpful when there is likely to be less health professional contact and was agreed by both panels as being important and relevant.

Third, our expert panel was comprised of consultant anaesthetists with trust-level quality improvement roles only. Within the UK, most pain management over the perioperative period is delivered by anaesthetic teams and acute pain teams comprised of clinical specialist nurses. Involvement of other members of the healthcare team, such as surgeons and specialist colorectal nurses, may have led to different results and support with future implementation into routine care.

The number of quality indicators in healthcare systems has been reported as an increasing burden, rather than a useful tool in achieving safer and better care.^33^ Our systematic process defined both the target audience who will operationalise the indicators in practice and involved patients to ensure the indicators developed measure dimensions of quality of care that are most important to them. This process led to a small number of indicators that reflected the panels’ views on importance but also actionability. The involvement of anaesthetic quality improvement leads in identifying barriers and toolkit resources aims to increase adoption and create an implementation climate at sites and increase willingness to participate in quality improvement in this field. We aim to continue to add further resources to the toolkit as new supportive materials are developed and barriers to implementation change.

The indicators and toolkit are the start of the quality improvement process in opioid stewardship in colorectal cancer surgery. The toolkit will support implementation and healthcare teams to identify barriers within their own settings and tailor strategies accordingly. Further work will determine if the introduction of the toolkit supports changes in practice and improves patient care.

## Supplementary material

10.1136/bmjopen-2024-092675 online supplemental file 1

10.1136/bmjopen-2024-092675 online supplemental file 2

10.1136/bmjopen-2024-092675 online supplemental file 3

10.1136/bmjopen-2024-092675 online supplemental file 4

10.1136/bmjopen-2024-092675 online supplemental file 5

10.1136/bmjopen-2024-092675 online supplemental file 6

10.1136/bmjopen-2024-092675 online supplemental file 7

## Data Availability

Data are available upon reasonable request.
