# Supplementary material for: Development of actionable quality indicators and an implementation toolkit for perioperative opioid stewardship in colorectal cancer in the UK Yorkshire and Humber region: a modified RAND consensus study
Source: BMJ Open. 2025 Sep 30;15(9):e092675. doi: 10.1136/bmjopen-2024-092675 (PMC12506214; doi:10.1136/bmjopen-2024-092675)
Supplement: online supplemental file 2 [file bmjopen-15-9-s002.docx]

**YORKSHIRE CANCER RESEARCH BOWEL CANCER IMPROVEMENT PROGRAMME**

**DEVELOPMENT OF ACTIONABLE QUALITY INDICATORS AND AN ACTION IMPLEMENTATION TOOLBOX TO REDUCE OPIOID-RELATED HARM IN SURGERY: A MODIFIED-RAND DELPHI STUDY**

**Round 2 – Online Survey**

**Introduction**

There is increasing attention being paid to the role of post-operative opioids in slowing recovery from surgery and contributing to long-term opioid use. The Yorkshire Cancer Research Bowel Cancer Improvement Programme (funded by Yorkshire Cancer Research) aims to improve outcomes for patients in Yorkshire and Humber with bowel cancer. A requirement for an effective opioid stewardship program is the ability to measure the appropriateness of opioid use.

Quality indicators are defined as measurable elements designed to evaluate aspects of quality of care. Currently there are no recommended quality indicators for opioid use in bowel cancer surgery. This project aims to develop these from existing literature and expert and local health care provider and support Trusts in implementing best practices. The indicators will then be used to measure current best practice within participating Trusts to improve opioid stewardship and patient outcomes following bowel cancer surgery.

In Round 1 we asked you for suggestions of potential quality indicators in opioid stewardship undertook a systematic literature review to identify potential quality indicators and identified guidelines regarding opioid stewardship. We now present you the full list and ask you to appraise each indicator using a 9-point Likert scale (1 = totally disagree, 9 = totally agree) on two criteria: 1) relevance, the impact of the indicator on opioid stewardship and 2) actionability, the extent to which an indicator offers direction for improvement in clinical practice.

Indicators with an overall median score between 4–9 on both relevance and actionability will be defined as potentially suitable and indicators with an overall median score between 1 and 4 will be defined as not suitable. We will select all potentially suitable indicators for the expert panel meeting (Round 4).

If you have any questions regarding this process please contact: Hannah Rossington, YCRBCIP Project Manager (email: [H.L.Rossington@leeds.ac.uk](mailto:H.L.Rossington@leeds.ac.uk)).

Thank you for your time,

Dr Sarah Alderson

Implementation Lead for YCR BCIP

**Page 1 - Demographic details:**

Name:

Role:

Trust:

**Page 2 – Potential opioid stewardship quality indictors**

**Section 1: Pre-assessment**

Quality Indicator 1 (Example): Documented pain history and evaluation, including current medication and non-prescription medicines

Relevance (impact of the indicator on opioid stewardship)

| 1 | 2 | 3 | 4 | 5 | 6 | 7 | 8 | 9 |  |
| --- | --- | --- | --- | --- | --- | --- | --- | --- | --- |
| Not relevant to opioid stewardship in bowel cancer surgery | | | Neither relevant nor not relevant | | | Relevant to opioid stewardship in bowel cancer surgery | | | Unable to score |

Actionability (the extent to which an indicator offers direction for improvement in clinical practice)

| 1 | 2 | 3 | 4 | 5 | 6 | 7 | 8 | 9 |  |
| --- | --- | --- | --- | --- | --- | --- | --- | --- | --- |
| Not likely to improve opioid stewardship in bowel cancer surgery | | | Neither likely nor not likely | | | Highly likely to opioid stewardship in bowel cancer surgery | | | Unable to score |

Comments: [free text]

[The above example will then be repeated for each quality indicator identified]

**Final page:**

Thank you for completing the survey. Once we have collated and analysed these results, we will be in touch to arrange Round 4 of the modified RAND consensus process as a meeting.
