# Supplementary material for: Development of actionable quality indicators and an implementation toolkit for perioperative opioid stewardship in colorectal cancer in the UK Yorkshire and Humber region: a modified RAND consensus study
Source: BMJ Open. 2025 Sep 30;15(9):e092675. doi: 10.1136/bmjopen-2024-092675 (PMC12506214; doi:10.1136/bmjopen-2024-092675)
Supplement: online supplemental file 3 [file bmjopen-15-9-s003.pdf]

BCIP Patient Panel

Development of Opioid Indicators for Bowel Cancer Surgery

Yorkshire Cancer Research
UNIVERSITY OF LEEDS

1

PPI Panel Plan

- Aim of project
- Research plan
- How potential indicators were identified
- Patient journey for bowel cancer surgery
- Types of indicators
- Scoring of indicators

Yorkshire Cancer Research
UNIVERSITY OF LEEDS

2

Aim of project

- Opioids are painkillers that are frequently prescribed, especially after surgery
- Taking opioids may increase recovery time and can lead to opioid dependence
- Quality indicators measure the quality-of-care patients receive
- There are no quality indicators for opioid use in bowel cancer surgery

Yorkshire Cancer Research
UNIVERSITY OF LEEDS

3

Research Plan

1. Identification of potential indicators from literature, guidelines and expert suggestions
2. Expert online survey to rank relevance and actionability
3. Patient panel to rank according to importance
4. Expert panel to meet decide on final indicators
5. Develop 'toolkit' to help Trusts achieve quality indicators

Yorkshire Cancer Research
UNIVERSITY OF LEEDS

4

How potential indicators were identified

- Literature review
- Existing guidelines
- Expert panel suggestion

= **73** indicators identified

Yorkshire Cancer Research
UNIVERSITY OF LEEDS

5

Patient Journey through Bowel Cancer Surgery

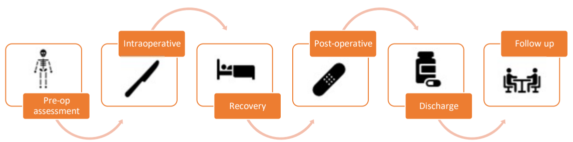

Yorkshire Cancer Research
UNIVERSITY OF LEEDS

6

Types of Indicators

PROCESS

OUTCOME

STRUCTURAL

Scoring of indicators

- 69 indicators remain after Round 2
- 4 indicators did not reach criteria for further inclusion

- 5 minutes per indicator
- Discuss indicator and score (1-9 with 9 being most important)

Pre-operative Indicators

1. Discussion with patient regarding realistic expectations of pain post-op, that includes goal to get DREAMing (DRinking, EAting and Mobilisation)

- Process indicator
- Identified from literature, guidelines and panel suggestion

- Expert score:
  - Relevance = 8
  - Actionability = 8

- RANK = 10th

2. Patient informed of risks of opioid medication, that post-op opioids will be a short course and deprescribed.

- Process indicator
- Identified from literature, guidelines and panel suggestion

- Expert panel:
  - Relevance = 7
  - Actionability = 7

  - RANK = 45

3. Patient provided educational materials on pain, including a documented pain management plan

- Process indicator
- Identified from literature, guidelines and panel suggestion

- Expert panel:
  - Relevance = 8
  - Actionability = 8

  - Rank = 12
