# Supplementary material for: Development of actionable quality indicators and an implementation toolkit for perioperative opioid stewardship in colorectal cancer in the UK Yorkshire and Humber region: a modified RAND consensus study
Source: BMJ Open. 2025 Sep 30;15(9):e092675. doi: 10.1136/bmjopen-2024-092675 (PMC12506214; doi:10.1136/bmjopen-2024-092675)
Supplement: online supplemental file 4 [file bmjopen-15-9-s004.docx]

BCIP Anaesthetics Opioid quality indicators consensus rounds 2 and 3, January 2023

# Background

There is increasing attention being paid to the role of post-operative opioids in slowing recovery from surgery and contributing to long-term opioid use. The Yorkshire Cancer Research Bowel Cancer Improvement Programme (funded by Yorkshire Cancer Research) aims to improve outcomes for patients in Yorkshire and Humber with bowel cancer. A requirement for an effective opioid stewardship program is the ability to measure the appropriateness of opioid use.

Quality indicators are defined as measurable elements designed to evaluate aspects of quality of care. Currently there are no recommended quality indicators for opioid use in bowel cancer surgery. This project aims to develop these from existing literature and expert and local health care provider and support Trusts in implementing best practices. The indicators will then be used to measure current best practice within participating Trusts to improve opioid stewardship and patient outcomes following bowel cancer surgery.

# Participants

## Location

| The Rotherham NHSFT |
| --- |
| Leeds Teaching Hospitals NHS Trust x 2 |
| York |
| Hull University Teaching Hospitals NHS Trust |
| Sheffield Teaching Hospitals |
| West Suffolk |
| CHFT |
| Chesterfield |
| Northern Lincolnshire and Goole NHS Foundation Trust |
| Patients: 6 members of BCIP PPIE group.  4 members completed the scoring during an online meeting on 13^th^ October 2022, 2 members completed via emailed survey. |

# Criteria for inclusion/exclusion to round 4

Relevance = the impact of the indicator on opioid stewardship

Actionability = the extent to which an indicator offers direction for improvement in clinical practice and achievement is under the control of the clinician.

Importance = how significant patients feel that achieving this indicator will improve their care

Indicators with a median score between 5–9 on both relevance and actionability (expert panel) and importance (patient panel) will be defined as potentially suitable and highlighted in blue.

Indicators with a median score between 1 and 4 will be defined as not suitable and highlighted in yellow.

# Pre-Operative Indicators

## Patient Education

### Quality Indicator 1:

Discussion with patient regarding realistic expectations of pain post-op, that includes goal to get DREAMing (DRinking, EAting and Mobilisation)

Process indicator - identified from literature, guidelines and panel suggestion

|  | 0-4 % | 5-9 % | Median Score |
| --- | --- | --- | --- |
| Relevance | 0 | 100 | 8 |
| Actionability | 0 | 100 | 8 |
| Importance | 0 | 100 | 8 |

Rank = 10 (experts), =12 (patients)

### Quality Indicator 2:

Patient informed of risks of opioid medication, that post-op opioids will be a short course and deprescribed.

Process indicator - identified from literature, guidelines and panel suggestion

|  | 0-4 % | 5-9 % | Median Score |
| --- | --- | --- | --- |
| Relevance | 10 | 90 | 7 |
| Actionability | 0 | 100 | 7 |
| Importance | 0 | 100 | 7 |

Rank = 45 (expert), = 35 (patientsl)

### Quality Indicator 3:

Patient provided educational materials on pain, including a documented pain management plan.

Process indicator - identified from literature, guidelines and panel suggestion

|  | 0-4 % | 5-9 % | Median Score |
| --- | --- | --- | --- |
| Relevance | 0 | 100 | 8 |
| Actionability | 10 | 90 | 8 |
| Importance | 50 | 50 | 5 |

Rank = 12 (expert), 73 (patient)

## Staff Education

### Quality Indicator 4:

Presence of multi-professional education materials for staff on opioid stewardship and need for multi-modal analgesia.

Structural indicator - identified from literature

|  | 0-4 % | 5-9 % | Median Score |
| --- | --- | --- | --- |
| Relevance | 0 | 100 | 9 |
| Actionability | 0 | 100 | 8 |
| Importance | 0 | 100 | 9 |

Rank = 1 (expert), =1 (patient)

## Preoperative Patient Optimisation

### Quality Indicator 5:

Identification of pre-operative use of opioid medications, including Daily Morphine Equivalent Dose.

Process indicator - identified from literature and panel suggestion

|  | 0-4 % | 5-9 % | Median Score |
| --- | --- | --- | --- |
| Relevance | 0 | 100 | 8 |
| Actionability | 10 | 90 | 7 |
| Importance | 17 | 83 | 7 |

Rank = 24 (expert), 35 (patient)

### Quality Indicator 6:

Referral to specialist pain service for opioid weaning and optimisation and perioperative analgesic planning in patients with potential opioid tolerance or complex pain needs.

Process indicator - identified from literature, guidelines and panel suggestion

|  | 0-4 % | 5-9 % | Median Score |
| --- | --- | --- | --- |
| Relevance | 10 | 90 | 8 |
| Actionability | 40 | 60 | 5 |
| Importance | 0 | 100 | 6 |

Rank = 40 (expert), 56 (patient)

### Quality Indicator 7:

Referral for counselling or psychosocial support for patients with complex pain needs.

Process indicator - identified from literature and guidelines

|  | 0-4 % | 5-9 % | Median Score |
| --- | --- | --- | --- |
| Relevance | 10 | 90 | 7 |
| Actionability | 70 | 30 | 4 |
| Importance | 0 | 100 | 8 |

Rank = 62 (expert), = 27 (patients)

### Quality Indicator 8:

Opioid Risk Tool (ORT) used to identify preoperatively patients at greater risk of persistent postoperative opioid use (PPOU).

Process indicator - identified from literature, guidelines and panel suggestion

|  | 0-4 % | 5-9 % | Median Score |
| --- | --- | --- | --- |
| Relevance | 0 | 100 | 7 |
| Actionability | 0 | 100 | 6 |
| Importance | 17 | 83 | 6 |

Rank = 51 (expert), 65 (patient)

### Quality Indicator 9:

Biopsychosocial assessment of pain and history of use of analgesic medications including opioids.

Process indicator - identified from literature, guidelines and panel suggestion

|  | 0-4 % | 5-9 % | Median Score |
| --- | --- | --- | --- |
| Relevance | 0 | 100 | 6 |
| Actionability | 30 | 70 | 5 |
| Importance | 0 | 100 | 6 |

Rank = 67 (experts), 65 (patients)

### Quality Indicator 10:

Screening tool used to identify preoperatively patients at greater risk of postoperative Opioid Related Adverse Drug Events (ORADE’s).

Process indicator - identified from literature and guidelines

|  | 0-4 % | 5-9 % | Median Score |
| --- | --- | --- | --- |
| Relevance | 0 | 100 | 7 |
| Actionability | 10 | 90 | 7 |
| Importance | 0 | 100 | 6 |

Rank = 42 (experts), 56 (patients)

### Quality Indicator 11:

Wean preoperative opioids to target of 60mg Morphine Equivalent Dose or below and by no more than 10% per week.

Process indicator - identified from guidelines

|  | 0-4 % | 5-9 % | Median Score |
| --- | --- | --- | --- |
| Relevance | 20 | 80 | 6 |
| Actionability | 80 | 20 | 4 |
| Importance | 17 | 83 | 5 |

Rank = 72 (experts), 70 (patients)

## Patient or Procedure Specific Prescribing or Deprescribing

### Quality Indicator 12:

Presence of a protocol to reduce perioperative opioid use with preoperative multimodal analgesia, including adjuncts such as NSAID’s and medications that may limit pain experience such as antiemetics.

Structural indicator - identified from literature and panel suggestion

|  | 0-4 % | 5-9 % | Median Score |
| --- | --- | --- | --- |
| Relevance | 0 | 100 | 8 |
| Actionability | 0 | 100 | 8 |
| Importance | 0 | 100 | 7 |

Rank = 10 (experts), 35 (patients)

### Quality Indicator 13:

Documentation of ‘universal precautions’ when initiating perioperative opioids.

Process indicator - identified from literature and panel suggestion

|  | 0-4 % | 5-9 % | Median Score |
| --- | --- | --- | --- |
| Relevance | 10 | 90 | 7 |
| Actionability | 10 | 90 | 6 |
| Importance | 0 | 100 | 7 |

Rank = 52 (experts), 35 (patients)

### Quality Indicator 14:

Opioid medications not increased preoperatively.

Process indicator - identified from literature and panel suggestion

|  | 0-4 % | 5-9 % | Median Score |
| --- | --- | --- | --- |
| Relevance | 0 | 100 | 7 |
| Actionability | 50 | 50 | 5 |
| Importance | 0 | 100 | 6 |

Rank = 57 (experts), 56 (patients)

### Quality Indicator 15:

Individualised perioperative pain management plan communicated to surgical, anaesthetic, care of elderly and frailty teams.

Process indicator - identified from literature, guidelines and panel suggestion

|  | 0-4 % | 5-9 % | Median Score |
| --- | --- | --- | --- |
| Relevance | 0 | 100 | 7 |
| Actionability | 40 | 60 | 5 |
| Importance | 0 | 100 | 7 |

Rank = 56 (experts), 35 (patients)

## Comments:

Experts:

- Some very worthwhile indicators but will be difficult to action
- I think lots of good quality indicators, not sure how actionable they are from a periop team perspective
- The responses are applicable to patients undergoing expedited cancer surgery. Preop weaning of opioids is of proven benefit to patients undergoing arthroplasty surgery, but it may be a bridge too far in patients awaiting urgent cancer surgery

Patients:

- As a patient it is helpful to know the recovery plan and importance of it. I don't remember this being discussed with me. (indicator 1)
- I understand why this is important but surely the primary purpose of pain relief is more so. I don't remember this being discussed with me. (indicator 2)
- I don't remember this being discussed with me. (indicator 3)
- I can see how this is very important for some people (indicator 7)
- must have training and protocol - won't work if one without the other (indicator 12)
- I recall my pain was increasing pre-treatment so I think this may not be practical (indicator 14)
- Important for frail elderly patients (indicator 15)

# Intraoperative Indicators

## Patient or Procedure Specific Prescribing or Deprescribing

### Quality Indicator 16:

Presence of an opioid-sparing protocol for intra operative use which includes minimally invasive surgery, regional blocks, neuraxial techniques and multimodal analgesia.

Structural indicator - identified from literature, guidelines and panel suggestion

|  | 0-4 % | 5-9 % | Median Score |
| --- | --- | --- | --- |
| Relevance | 0 | 100 | 9 |
| Actionability | 0 | 100 | 8 |
| Importance | 0 | 100 | 9 |

Rank = 1 (experts), 5 (patients)

### Quality Indicator 17:

Adherence to intraoperative opioid-sparing protocol with documented use of multimodal approach which includes minimally invasive surgery, regional blocks, neuraxial techniques, non-pharmacological and multimodal analgesia.

Structural indicator - identified from literature, guidelines and panel suggestion

|  | 0-4 % | 5-9 % | Median Score |
| --- | --- | --- | --- |
| Relevance | 0 | 100 | 8 |
| Actionability | 10 | 90 | 8 |
| Importance | 0 | 100 | 8 |

Rank = 12 (experts), 12 (patients)

### Quality Indicator 18:

Use of procedure-related PROSPECT recommendations for analgesia.

Process indicator - identified from guidelines

|  | 0-4 % | 5-9 % | Median Score |
| --- | --- | --- | --- |
| Relevance | 10 | 90 | 8 |
| Actionability | 30 | 70 | 6 |
| Importance | 0 | 100 | 7 |

Rank = 37 (expert), 35 (patients)

### Quality Indicator 19:

A documented individualised intraoperative plan for patients already taking opioid medications, including use of regional, neuraxial techniques and non-opioid adjuncts as recommended by ANZCA FPM, with planned management of acute pain.

Process indicator - identified from guidelines and panel suggestion

|  | 0-4 % | 5-9 % | Median Score |
| --- | --- | --- | --- |
| Relevance | 0 | 100 | 8 |
| Actionability | 20 | 80 | 7 |
| Importance | 0 | 100 | 8 |

Rank = 27 (experts), 27 (patients)

## Comments:

Experts:

- I don't think there is prospect advice for lap bowel work - and that's the majority of what we do.
- we can make massive impact to outcomes by utilising anaesthetic/ analgesic techniques that do not tether the patient to the bed post-operatively

Patients:

# Recovery Indicators

## Patient or Procedure Specific Prescribing or Deprescribing

### Quality Indicator 20:

Patient reviewed in PACU for new risk factors for PPOU, including formation of a stoma.

Process indicator - identified from literature

|  | 0-4 % | 5-9 % | Median Score |
| --- | --- | --- | --- |
| Relevance | 10 | 90 | 6 |
| Actionability | 30 | 70 | 5 |
| Importance | 0 | 100 | 6 |

Rank = 69 (experts), 56 (patients)

### Quality Indicator 21:

Presence of an opioid-sparing protocol for recovery/immediate postoperative use which includes regional blocks, non-pharmacological treatments, standardized rescue, multimodal analgesia and avoidance of PCA and PCEAs if able to take oral fluids and analgesia.

Structural indicator - identified from literature and panel suggestion

|  | 0-4 % | 5-9 % | Median Score |
| --- | --- | --- | --- |
| Relevance | 10 | 90 | 9 |
| Actionability | 20 | 80 | 7 |
| Importance | 0 | 100 | 8 |

Rank = 8 (experts), 12 (patients)

### Quality Indicator 22:

Adherence to recovery/immediate postoperative opioid-sparing protocol, including continuing multimodal analgesia, avoiding opioid boluses, and simple analgesics.

Process indicator - identified from literature and panel suggestion

|  | 0-4 % | 5-9 % | Median Score |
| --- | --- | --- | --- |
| Relevance | 10 | 90 | 8 |
| Actionability | 40 | 60 | 6 |
| Importance | 0 | 100 | 7 |

Rank = 39 (experts), 35 (patients)

### Quality Indicator 23:

Functional assessment of pain before leaving PACU which includes ability to cough and deep breathe.

Process indicator - identified from guidelines and panel suggestion

|  | 0-4 % | 5-9 % | Median Score |
| --- | --- | --- | --- |
| Relevance | 0 | 100 | 8 |
| Actionability | 10 | 90 | 8 |
| Importance | 0 | 100 | 9 |

Rank = 12 (experts), 5 (patients)

### Quality Indicator 24:

Assessment of sedation in PACU.

Process indicator - identified from panel suggestion

|  | 0-4 % | 5-9 % | Median Score |
| --- | --- | --- | --- |
| Relevance | 20 | 80 | 8 |
| Actionability | 10 | 90 | 8 |
| Importance | 0 | 100 | 8 |

Rank = 18 (experts), 12 (patients)

## Comments:

Experts:

- I'm not sure sedation increases risk of post op opioid consumption. I think using blocks as rescue for abdo surgery unlikely to be a good quality indicator

Patients:

# Postoperative Indicators

## Patient education

### Quality Indicator 25:

Patient given educational materials that emphasise the need and benefit of non-opioid non-pharmacological approaches to analgesia.

Process indicator - identified from guidelines

|  | 0-4 % | 5-9 % | Median Score |
| --- | --- | --- | --- |
| Relevance | 0 | 100 | 8 |
| Actionability | 0 | 100 | 7 |
| Importance | 0 | 100 | 6 |

Rank = 19 (experts), 65 (patients)

## Patient or Procedure Specific Prescribing or Deprescribing

### Quality Indicator 26:

Presence of a referral route to an acute pain service that includes pathways for opioid tolerant patients and readmissions due to pain or opioids.

Structural indicator - identified from literature and guidelines

|  | 0-4 % | 5-9 % | Median Score |
| --- | --- | --- | --- |
| Relevance | 0 | 100 | 8 |
| Actionability | 10 | 90 | 8 |
| Importance | 0 | 100 | 8 |

Rank = 12 (experts), 12 (patients)

### Quality Indicator 27:

Daily postoperative pain review.

Process indicator - identified from literature

|  | 0-4 % | 5-9 % | Median Score |
| --- | --- | --- | --- |
| Relevance | 0 | 100 | 9 |
| Actionability | 20 | 80 | 7 |
| Importance | 0 | 100 | 9 |

Rank = 7 (experts), 5 (patients)

### Quality Indicator 28:

Presence of a postoperative opioid-sparing protocol which includes regional blocks, non-pharmacological approaches, simple analgesia and multimodal analgesia, with oral medications prioritised, particularly those that can be administered by one nurse (oramorph rather than oxycodone), avoidance of long-acting and im opioids and prescribed according to renal function and age.

Structural indicator - identified from literature, guidelines and panel suggestion

|  | 0-4 % | 5-9 % | Median Score |
| --- | --- | --- | --- |
| Relevance | 0 | 100 | 8 |
| Actionability | 0 | 100 | 7 |
| Importance | 0 | 100 | 8 |

Rank = 19 (experts), 12 (patients)

### Quality Indicator 29:

Adherence to a postoperative opioid-sparing protocol.

Process indicator - identified from literature

|  | 0-4 % | 5-9 % | Median Score |
| --- | --- | --- | --- |
| Relevance | 0 | 100 | 8 |
| Actionability | 0 | 100 | 7 |
| Importance | 0 | 100 | 8 |

Rank = 19 (experts), 12 (patients)

### Quality Indicator 30:

Presence of protocol for patients already taking opioids that includes pain reviews, lowest effective dose, avoidance of escalation of opioids postoperatively and use of non-opioid analgesia first-line.

Process indicator - identified from guidelines

|  | 0-4 % | 5-9 % | Median Score |
| --- | --- | --- | --- |
| Relevance | 0 | 100 | 8 |
| Actionability | 0 | 100 | 7 |
| Importance | 0 | 100 | 8 |

Rank = 19 (experts), 12 (patients)

### Quality Indicator 31:

Maximum daily oral MME for an opioid-naïve patient of 50mg.

Process indicator - identified from guidelines

|  | 0-4 % | 5-9 % | Median Score |
| --- | --- | --- | --- |
| Relevance | 10 | 90 | 7 |
| Actionability | 20 | 80 | 5 |
| Importance | 33 | 67 | 5 |

Rank = 58 (experts), 70 (patients)

### Quality Indicator 32:

Pharmacist or Pain team review of opiate prescribing if greater than 3 day use postoperatively.

Process indicator - identified from panel suggestion

|  | 0-4 % | 5-9 % | Median Score |
| --- | --- | --- | --- |
| Relevance | 10 | 90 | 8 |
| Actionability | 10 | 90 | 7 |
| Importance | 0 | 100 | 7 |

Rank = 32 (experts), 35 (patients)

### Quality Indicator 33:

Patients receiving postoperative opioids have sedation score documented.

Process indicator - identified from literature and guidelines

|  | 0-4 % | 5-9 % | Median Score |
| --- | --- | --- | --- |
| Relevance | 10 | 90 | 9 |
| Actionability | 10 | 90 | 8 |
| Importance | 0 | 100 | 9 |

Rank = 4 (experts), 1 (patients)

### Quality Indicator 34:

Rate of ORADEs including severity and impact on length of stay.

Outcome indicator - identified from literature and panel suggestion

|  | 0-4 % | 5-9 % | Median Score |
| --- | --- | --- | --- |
| Relevance | 10 | 90 | 8 |
| Actionability | 10 | 90 | 6 |
| Importance | 0 | 100 | 8 |

Rank = 35 (experts), 27 (patients)

## Comments:

Experts:

- The use of a limit of MME probably has limited value, young people may require more, and the elderly may be harmed. I think limiting opioids to 3 days will swing the pendulum towards opioid free, which is not the way forward. I would also suggest continued use of NPS to assess pain trajectory to pick up transition to chronic pain , and also surgical catastrophes

Patients:

- Patients noted that staffing meant 2 nurse sign-off was not easy in hospital so important to have one nurse sign-off (indicator 28)
- Seems somewhat arbitrary (indicator 31)

# Discharge Indicators

## Patient Education

### Quality Indicator 35:

Patient given education leaflet on safe administration, storage, weaning and disposal of unused opioids and avoidance of opioid diversion.

Process indicator - identified from literature and guidelines

|  | 0-4 % | 5-9 % | Median Score |
| --- | --- | --- | --- |
| Relevance | 10 | 90 | 8 |
| Actionability | 20 | 80 | 8 |
| Importance | 0 | 100 | 9 |

Rank = 17 (experts), 5 (patients)

### Quality Indicator 36:

Patient given opioid specific discharge advice including not driving for up to 4 weeks until opioid dose is stable and managing post-operative pain.

Process indicator - identified from literature and panel suggestion

|  | 0-4 % | 5-9 % | Median Score |
| --- | --- | --- | --- |
| Relevance | 0 | 100 | 8 |
| Actionability | 20 | 80 | 7 |
| Importance | 0 | 100 | 8 |

Rank = 27 (experts), 12 (patients)

### Quality Indicator 37:

Patient education documented on pain management for mobilisation with deprescribing order: opioid, then NSAID, then paracetamol, with advice on drowsiness or worsening pain.

Process indicator - identified from guidelines and panel suggestion

|  | 0-4 % | 5-9 % | Median Score |
| --- | --- | --- | --- |
| Relevance | 0 | 100 | 8 |
| Actionability | 10 | 90 | 6 |
| Importance | 0 | 100 | 8 |

Rank = 33 (experts), 27 (patients)

### Quality Indicator 38:

Patient given BPS leaflet on managing post-operative pain.

Process indicator - identified from panel suggestion

|  | 0-4 % | 5-9 % | Median Score |
| --- | --- | --- | --- |
| Relevance | 0 | 100 | 7 |
| Actionability | 20 | 80 | 7 |
| Importance | 17 | 83 | 7 |

Rank = 44 (experts), 52 (patients)

### Quality Indicator 39:

Patient given point of contact for ongoing pain issue.

Process indicator - identified from panel suggestion

|  | 0-4 % | 5-9 % | Median Score |
| --- | --- | --- | --- |
| Relevance | 0 | 100 | 9 |
| Actionability | 0 | 100 | 8 |
| Importance | 0 | 100 | 9 |

Rank = 1 (experts), 1 (patients)

## Patient or Procedure Specific Prescribing or Deprescribing

### Quality Indicator 40:

Presence of a patient group specific protocol for discharge opioid prescribing that includes calculating dose for discharge based upon past 24-hour use of opioids, recommends using lowest dose of opioids possible for the shortest duration, opioids and non-opioids to be prescribed separately.

Structural indicator - identified from literature and guidelines

|  | 0-4 % | 5-9 % | Median Score |
| --- | --- | --- | --- |
| Relevance | 0 | 100 | 7 |
| Actionability | 10 | 90 | 7 |
| Importance | 0 | 100 | 7 |

Rank = 42 (experts), 35 (patients)

### Quality Indicator 41:

Presence of a de-escalation plan for opioids prescribed on discharge, including a tapering plan for those taking >= 50mg MME for >= 3 weeks or no tapering if opioid naive and opioids are ceased once functional recovery is achieved.

Structural indicator - identified from literature and guidelines

|  | 0-4 % | 5-9 % | Median Score |
| --- | --- | --- | --- |
| Relevance | 0 | 100 | 8 |
| Actionability | 10 | 90 | 7 |
| Importance | 0 | 100 | 7 |

Rank = 24 (experts), 35 (patients)

### Quality Indicator 42:

Electronic clinical quality measure (eCQM) to assess potentially inappropriate high dose postoperative opioid prescribing practices e.g an average daily dose ≥90 MME for the duration of postoperative opioid prescription in preoperatively opioid naïve patients.

Structural indicator - identified from literature

|  | 0-4 % | 5-9 % | Median Score |
| --- | --- | --- | --- |
| Relevance | 10 | 90 | 8 |
| Actionability | 30 | 70 | 6 |
| Importance | 0 | 100 | 8 |

Rank = 37 (experts), 27 (patients)

### Quality Indicator 43:

Procedure specific post op prescribing guidelines to provide enough doses to cover 75% of patients.

Structural indicator - identified from literature

|  | 0-4 % | 5-9 % | Median Score |
| --- | --- | --- | --- |
| Relevance | 10 | 90 | 7 |
| Actionability | 30 | 70 | 7 |
| Importance | 0 | 100 | 6 |

Rank = 50 (experts), 56 (patients)

### Quality Indicator 44:

Procedure specific prescribing limits built into electronic patient record.

Structural indicator - identified from literature

|  | 0-4 % | 5-9 % | Median Score |
| --- | --- | --- | --- |
| Relevance | 10 | 90 | 7 |
| Actionability | 30 | 70 | 5 |
| Importance | 17 | 83 | 6 |

Rank = 59 (experts), 56 (patients)

### Quality Indicator 45:

Pain management plan and tapering strategies, including dose, amount supplied, and duration of 5 days and no longer than 7 days, clearly communicated to primary care team in a timely manner.

Process indicator - identified from literature, guidelines and panel suggestion

|  | 0-4 % | 5-9 % | Median Score |
| --- | --- | --- | --- |
| Relevance | 0 | 100 | 8 |
| Actionability | 20 | 80 | 7 |
| Importance | 0 | 100 | 8 |

Rank = 27 (experts), 12 (patients)

### Quality Indicator 46:

Opioid present on hospital discharge prescription.

Process indicator - identified from literature

|  | 0-4 % | 5-9 % | Median Score |
| --- | --- | --- | --- |
| Relevance | 10 | 90 | 7 |
| Actionability | 20 | 80 | 7 |
| Importance | 0 | 100 | 7 |

Rank = 47 (experts), 35 (patients)

### Quality Indicator 47:

Procedure-specific mean discharge MME prescribed.

Process indicator - identified from literature

|  | 0-4 % | 5-9 % | Median Score |
| --- | --- | --- | --- |
| Relevance | 0 | 100 | 6 |
| Actionability | 20 | 80 | 5 |
| Importance | 0 | 100 | 6 |

Rank = 66 (experts), 56 (patients)

### Quality Indicator 48:

Frequency of slow-release opioids prescribed on discharge.

Process indicator - identified from literature

|  | 0-4 % | 5-9 % | Median Score |
| --- | --- | --- | --- |
| Relevance | 10 | 90 | 7 |
| Actionability | 10 | 90 | 7 |
| Importance | 0 | 100 | 7 |

Rank = 46 (experts), 35 (patients)

### Quality Indicator 49:

Frequency of immediate-release opioids prescribed on discharge.

Process indicator - identified from literature

|  | 0-4 % | 5-9 % | Median Score |
| --- | --- | --- | --- |
| Relevance | 10 | 90 | 7 |
| Actionability | 20 | 80 | 7 |
| Importance | 0 | 100 | 7 |

Rank = 47 (experts), 52 (patients)

### Quality Indicator 50:

Non-opioid adjuvant analgesia present on discharge prescription.

Process indicator - identified from literature

|  | 0-4 % | 5-9 % | Median Score |
| --- | --- | --- | --- |
| Relevance | 10 | 90 | 7 |
| Actionability | 20 | 80 | 7 |
| Importance | 0 | 100 | 7 |

Rank = 47 (experts), 35 (patients)

### Quality Indicator 51:

Opioids not prescribed for more than 3-7 days and do not include modified-release formulations.

Process indicator - identified from guidelines and panel suggestion

|  | 0-4 % | 5-9 % | Median Score |
| --- | --- | --- | --- |
| Relevance | 0 | 100 | 8 |
| Actionability | 0 | 100 | 7 |
| Importance | 0 | 100 | 7 |

Rank = 19 (experts), 35 (patients)

### Quality Indicator 52:

Patients should not be discharged on strong opioids.

Process indicator - identified from panel suggestion

|  | 0-4 % | 5-9 % | Median Score |
| --- | --- | --- | --- |
| Relevance | 40 | 60 | 7 |
| Actionability | 40 | 60 | 5 |
| Importance | 33 | 67 | 6 |

Rank = 61 (experts), 65 (patients)

### Quality Indicator 53:

Senior review of need for discharge opioid medication.

Process indicator - identified from panel suggestion

|  | 0-4 % | 5-9 % | Median Score |
| --- | --- | --- | --- |
| Relevance | 20 | 80 | 7 |
| Actionability | 50 | 50 | 5 |
| Importance | 0 | 100 | 7 |

Rank = 60 (experts), 35 (patients)

### Quality Indicator 54:

Presence of recording tool for opioids used during inpatient stay.

Process indicator - identified from literature

|  | 0-4 % | 5-9 % | Median Score |
| --- | --- | --- | --- |
| Relevance | 0 | 100 | 6 |
| Actionability | 20 | 80 | 6 |
| Importance | 0 | 100 | 7 |

Rank = 64 (experts), =52 (patients)

### Quality Indicator 55:

Use of ‘reverse pain ladder’ for de-escalation of opioids.

Process indicator - identified from literature

|  | 0-4 % | 5-9 % | Median Score |
| --- | --- | --- | --- |
| Relevance | 0 | 100 | 8 |
| Actionability | 10 | 90 | 8 |
| Importance | 0 | 100 | 8 |

Rank = 12 (experts), 12 (patients)

### Quality Indicator 56:

Total milligram of morphine equivalents (MME) consumed during 24h prior to discharge.

Outcome indicator - identified from literature

|  | 0-4 % | 5-9 % | Median Score |
| --- | --- | --- | --- |
| Relevance | 0 | 100 | 6 |
| Actionability | 20 | 80 | 6 |
| Importance | 0 | 100 | 6 |

Rank = 64 (experts), 56 (patients)

### Quality Indicator 57:

Total milligram of morphine equivalents (MME) consumed during hospital stay.

Outcome indicator - identified from literature

|  | 0-4 % | 5-9 % | Median Score |
| --- | --- | --- | --- |
| Relevance | 30 | 70 | 6 |
| Actionability | 40 | 60 | 5 |
| Importance | 17 | 83 | 6 |

Rank = 70 (experts), =65 (patients)

### Quality Indicator 58:

Procedure specific mean daily inpatient MME used.

Outcome indicator - identified from literature

|  | 0-4 % | 5-9 % | Median Score |
| --- | --- | --- | --- |
| Relevance | 20 | 80 | 5 |
| Actionability | 40 | 60 | 5 |
| Importance | 17 | 83 | 5 |

Rank = 73 (experts), = 70 (patients)

## Opioid Related Adverse Drug Events (ORADEs)

### Quality Indicator 59:

Identify those at risk of ORADEs when prescribing opioids for use at home. Male, obese, over 65, greater comorbidities, pre-op opioid use, concurrent sedative medication use.

Process indicator - identified from literature

|  | 0-4 % | 5-9 % | Median Score |
| --- | --- | --- | --- |
| Relevance | 0 | 100 | 8 |
| Actionability | 10 | 90 | 7 |
| Importance | 0 | 100 | 8 |

Rank = 24 (experts), = 12 (patients)

## Comments:

Experts:

- Lots of these seem very repetitive, I think the patients should get a deprescribing plan and a contact if they cannot achieve it. A lot of this could not be collected without dedicated resource so they are not that actionable... you could get the pre discharge opioid consumption but who would get it? No one has the time to wade through hospital IT
- in terms of OIVI, many instances occur in patients with no risk appreciated factors, thus the need for a blanket approach, rather than a targeted approach
- The use of the word frequency in some of the above indicators could have a no. of different meanings. Does frequency mean literally the no of times a day a patient should be directed to take their opiate (ie clarity of directions in prescription) or does it mean organisationally measuring the frequency in which opiates are prescribed by the department in cancer patients at discharge??

Patients:

- Leaflet needs to be explained by a health professional not just given to patient; From a patients point of view, this does appear to be lacking somewhat (indicator 35)
- Make sure this is clear how this links/ is different with general post-operative advice following bowel surgery (indicator 36)
- Link with other education elements - so one educational moment for the patient (indicator 37)
- Patients noted lack of availability of leaflets in hospitals; Leaflets are not universally helpful or used (indicator 38)
- Very important for patients to have a one-stop contact point for patients (wouldn't want another number for pain). Should be 24/7 (indicator 39)
- Need to be confident the GP is able to take over after the 7 days to make sure this is feasible (indicator 51)
- Would need definition of Senior (indicator 53)
- Why wouldn’t this be done already? (indicator 54)
- Again, isn't this done already? (indicator 57)
- Seems like an easy win (indicator 59)

# Follow Up

## Staff Education

### Quality Indicator 60:

Prescribers sent quarterly reports on their prescribing compared to guidelines.

Structural indicator - identified from literature

|  | 0-4 % | 5-9 % | Median Score |
| --- | --- | --- | --- |
| Relevance | 10 | 90 | 7 |
| Actionability | 60 | 40 | 3 |
| Importance | 33 | 67 | 8 |

Rank = 63 (experts), 27 (patients)

## Patient or Procedure Specific Prescribing or Deprescribing

### Quality Indicator 61:

Presence of process to assess opioids prescribed versus opioids used following surgical procedures to allow tailoring of opioid prescriptions to need for a patient group/specific procedure.

Structural indicator - identified from literature

|  | 0-4 % | 5-9 % | Median Score |
| --- | --- | --- | --- |
| Relevance | 0 | 100 | 6 |
| Actionability | 50 | 50 | 5 |
| Importance | 17 | 83 | 7 |

Rank = 68 (experts), 52 (patients)

### Quality Indicator 62:

No repeat prescriptions of opioids following discharge, with new modified-release opioids only to be started in conjunction with specialist referral.

Process indicator - identified from guidelines and panel suggestion

|  | 0-4 % | 5-9 % | Median Score |
| --- | --- | --- | --- |
| Relevance | 10 | 90 | 8 |
| Actionability | 20 | 80 | 6 |
| Importance | 0 | 100 | 8 |

Rank = 36 (experts), 27 (patients)

### Quality Indicator 63:

A patient still on opioids at 90 days to have review in primary or secondary care or pain service referral.

Process indicator - identified from guidelines and panel suggestion

|  | 0-4 % | 5-9 % | Median Score |
| --- | --- | --- | --- |
| Relevance | 0 | 100 | 9 |
| Actionability | 0 | 100 | 7 |
| Importance | 0 | 100 | 9 |

Rank = 5 (experts), = 5 (patients)

### Quality Indicator 64:

Screening to ensure those requesting repeat opioids or still taking opioids beyond 2 weeks post-op are reviewed.

Process indicator - identified from guidelines

|  | 0-4 % | 5-9 % | Median Score |
| --- | --- | --- | --- |
| Relevance | 0 | 100 | 9 |
| Actionability | 10 | 90 | 6 |
| Importance | 0 | 100 | 9 |

Rank = 9 (experts), = 5 (patients)

### Quality Indicator 65:

Reduction of unused opioid in the community.

Outcome indicator - identified from literature

|  | 0-4 % | 5-9 % | Median Score |
| --- | --- | --- | --- |
| Relevance | 10 | 90 | 8 |
| Actionability | 40 | 60 | 5 |
| Importance | 0 | 100 | 8 |

Rank = 40 (experts), =12 (patients)

### Quality Indicator 66:

Post operative prescription considered to have been given if opioids dispensed between 2-7 days following discharge.

Outcome indicator - identified from literature

|  | 0-4 % | 5-9 % | Median Score |
| --- | --- | --- | --- |
| Relevance | 10 | 90 | 6 |
| Actionability | 60 | 40 | 4 |
| Importance | 33 | 67 | 6 |

Rank = 71 (experts), 56 (patients)

### Quality Indicator 67:

Hospital analgesic policies include strategies to support post-discharge assessment and follow-up of patients at risk of becoming chronic opioid users.

Structural indicator - identified from literature

|  | 0-4 % | 5-9 % | Median Score |
| --- | --- | --- | --- |
| Relevance | 0 | 100 | 8 |
| Actionability | 30 | 70 | 6 |
| Importance | 17 | 83 | 8 |

Rank = 34 (experts), 12 (patients)

### Quality Indicator 68:

Presence of plan or protocol if opioid abuse or misuse is detected.

Structural indicator - identified from literature

|  | 0-4 % | 5-9 % | Median Score |
| --- | --- | --- | --- |
| Relevance | 0 | 100 | 8 |
| Actionability | 20 | 80 | 7 |
| Importance | 0 | 100 | 9 |

Rank = 27 (experts), 5 (patients)

### Quality Indicator 69:

Procedure for return and disposal of unused opioids.

Structural indicator - identified from guidelines and panel suggestion

|  | 0-4 % | 5-9 % | Median Score |
| --- | --- | --- | --- |
| Relevance | 0 | 100 | 9 |
| Actionability | 10 | 90 | 7 |
| Importance | 0 | 100 | 9 |

Rank = 6 (experts), 1(patients)

### Quality Indicator 70:

Use of higher dosage of opioids at any time (>50-60 MME).

Outcome indicator - identified from literature

|  | 0-4 % | 5-9 % | Median Score |
| --- | --- | --- | --- |
| Relevance | 20 | 80 | 7 |
| Actionability | 20 | 80 | 6 |
| Importance | 0 | 100 | 8 |

Rank = 55 (experts), 27 (patients)

### Quality Indicator 71:

PPOU: ongoing or increased (relative to pre-op) opioid use at 90-180/365 days post discharge.

Outcome indicator - identified from literature

|  | 0-4 % | 5-9 % | Median Score |
| --- | --- | --- | --- |
| Relevance | 10 | 90 | 7 |
| Actionability | 20 | 80 | 6 |
| Importance | 0 | 100 | 7 |

Rank = 53 (experts), 35 (patients)

### Quality Indicator 72:

Time to opioid cessation: a period without an opioid prescription equivalent to three times the estimated supply duration in preoperatively opioid naïve patients.

Outcome indicator - identified from literature

|  | 0-4 % | 5-9 % | Median Score |
| --- | --- | --- | --- |
| Relevance | 10 | 90 | 7 |
| Actionability | 30 | 70 | 6 |
| Importance | 0 | 100 | 7 |

Rank = 54 (experts), 35 (patients)

### Quality Indicator 73:

Incidence of opioid related re-admissions.

Outcome indicator - identified from literature

|  | 0-4 % | 5-9 % | Median Score |
| --- | --- | --- | --- |
| Relevance | 0 | 100 | 8 |
| Actionability | 20 | 80 | 7 |
| Importance | 0 | 100 | 8 |

Rank = 27 (experts), 12 (patients)

## Comments:

Experts:

- Again resources - it will be hard to track ongoing prescribing, obviously relevant but I am not sure actionable
- Standards 66 and 67 look the same
- Question 74 & 75 are the same

Patients:

- If not possible to be done at prescriber level patient feels it is important to do at department level. Don't want it dismissed as harder to action - this indicator was scored low (not to be taken forward) by expert panel (indicator 60)
- Importance of communication between primary and secondary care (indicator 62)
- Patients feel it is too vague. Should be a review by a specialised pain service not a GP (indicator 63)
- Would need to be more specific as to who would screen. Possibly at staff in follow-up appointment (indicator 64)
- Was a dicussion that some pharmacies are refusing medications being returned for disposal; No support to dispose of unused medication in my experience (indicator 65)
- Scored by patients but this indicator was identified by expert panel as to not go forward (low scoring) (indicator 66)
- Should be a shared care policy not a hospital policy - joint between primary and secondary care (indicator 67)
- Card for patients stating what they have been advised to do to avoid refusal of taking in medications to pharmacies etc (indicator 69)

# Summary Results

Results are sorted by expert ranking, followed by patient ranking. Results in *italics* did not reach criteria for taking forward to next round.

| Rank - Expert | Rank - Patient | Indicator Number | Indicator | Pt Stage | Category | Indicator Type | Relevance | 5-9% | 0-4% | Actionability | 5-9% | 0-4% | Importance | 5-9% | 0-4% |
| --- | --- | --- | --- | --- | --- | --- | --- | --- | --- | --- | --- | --- | --- | --- | --- |
| 1 | 1 | **4** | Presence of multi-professional education materials for staff on opioid stewardship and need for multi-modal analgesia. | Pre-Op | Staff Education | Process | **9** | 100 | 0 | **8** | 100 | 0 | **9** | 6 | 0 |
| 1 | 1 | **39** | Patient given point of contact for ongoing pain issue. | Discharge | Patient Education | Process | **9** | 100 | 0 | **8** | 100 | 0 | **9** | 6 | 0 |
| 1 | 5 | **16** | Presence of an opioid-sparing protocol for intra operative use which includes minimally invasive surgery, regional blocks, neuraxial techniques and multimodal analgesia. | Intraop | Pt or Pr Specific Px or DePx | Structural | **9** | 100 | 0 | **8** | 100 | 0 | **9** | 6 | 0 |
| 4 | 1 | **33** | Patients receiving postoperative opioids have sedation score documented. | Post Op | Pt or Pr Specific Px or DePx | Process | **9** | 90 | 10 | **8** | 90 | 10 | **9** | 6 | 0 |
| 5 | 5 | **63** | A patient still on opioids at 90 days to have review in primary or secondary care or pain service referral. | Follow Up | Pt or Pr Specific Px or DePx | Process | **9** | 100 | 0 | **7** | 100 | 0 | **9** | 6 | 0 |
| 6 | 1 | **69** | Procedure for return and disposal of unused opioids. | Follow Up | Pt or Pr Specific Px or DePx | Structural | **9** | 100 | 0 | **7** | 90 | 10 | **9** | 6 | 0 |
| 7 | 5 | **27** | Daily postoperative pain review. | Post Op | Pt or Pr Specific Px or DePx | Process | **9** | 100 | 0 | **7** | 80 | 20 | **9** | 6 | 0 |
| 8 | 12 | **21** | Presence of an opioid-sparing protocol for recovery/immediate postoperative use which includes regional blocks, non-pharmacological treatments, standardized rescue, multimodal analgesia and avoidance of PCA and PCEAs if able to take oral fluids and analgesia. | Recovery | Pt or Pr Specific Px or DePx | Structural | **9** | 90 | 10 | **7** | 80 | 20 | **8** | 6 | 0 |
| 9 | 5 | **64** | Screening to ensure those requesting repeat opioids or still taking opioids beyond 2 weeks post-op are reviewed. | Follow Up | Pt or Pr Specific Px or DePx | Process | **9** | 100 | 0 | **6** | 90 | 10 | **9** | 6 | 0 |
| 10 | 12 | **1** | Discussion with patient regarding realistic expectations of pain post-op, that includes goal to get DREAMing (DRinking, EAting and Mobilisation) | Pre-Op | Patient Education | Process | **8** | 100 | 0 | **8** | 100 | 0 | **8** | 6 | 0 |
| 10 | 35 | **12** | Presence of a protocol to reduce perioperative opioid use with preoperative multimodal analgesia, including adjuncts such as NSAID’s and medications that may limit pain experience such as antiemetics. | Pre-Op | Pt or Pr Specific Px or DePx | Structural | **8** | 100 | 0 | **8** | 100 | 0 | **7** | 6 | 0 |
| 12 | 5 | **23** | Functional assessment of pain before leaving PACU which includes ability to cough and deep breathe. | Recovery | Pt or Pr Specific Px or DePx | Process | **8** | 100 | 0 | **8** | 90 | 10 | **9** | 6 | 0 |
| 12 | 12 | **17** | Adherence to intraoperative opioid-sparing protocol with documented use of multimodal approach which includes minimally invasive surgery, regional blocks, neuraxial techniques, non-pharmacological and multimodal analgesia. | Intraop | Pt or Pr Specific Px or DePx | Structural | **8** | 100 | 0 | **8** | 90 | 10 | **8** | 6 | 0 |
| 12 | 12 | **26** | Presence of a referral route to an acute pain service that includes pathways for opioid tolerant patients and readmissions due to pain or opioids. | Post Op | Pt or Pr Specific Px or DePx | Structural | **8** | 100 | 0 | **8** | 90 | 10 | **8** | 6 | 0 |
| 12 | 12 | **55** | Use of ‘reverse pain ladder’ for de-escalation of opioids. | Discharge | Pt or Pr Specific Px or DePx | Process | **8** | 100 | 0 | **8** | 90 | 10 | **8** | 6 | 0 |
| 12 | 73 | **3** | Patient provided educational materials on pain, including a documented pain management plan. | Pre-Op | Patient Education | Structural | **8** | 100 | 0 | **8** | 90 | 10 | **5** | 3 | 3 |
| 17 | 5 | **35** | Patient given education leaflet on safe administration, storage, weaning and disposal of unused opioids and avoidance of opioid diversion. | Discharge | Patient Education | Process | **8** | 90 | 10 | **8** | 80 | 20 | **9** | 6 | 0 |
| 18 | 12 | **24** | Assessment of sedation in PACU. | Recovery | Pt or Pr Specific Px or DePx | Process | **8** | 80 | 20 | **8** | 90 | 10 | **8** | 6 | 0 |
| 19 | 12 | **28** | Presence of a postoperative opioid-sparing protocol which includes regional blocks, non-pharmacological approaches, simple analgesia and multimodal analgesia, with oral medications prioritised, particularly those that can be administered by one nurse (oramorph rather than oxycodone), avoidance of long-acting and im opioids and prescribed according to renal function and age. | Post Op | Pt or Pr Specific Px or DePx | Structural | **8** | 100 | 0 | **7** | 100 | 0 | **8** | 6 | 0 |
| 19 | 12 | **29** | Adherence to a postoperative opioid-sparing protocol. | Post Op | Pt or Pr Specific Px or DePx | Process | **8** | 100 | 0 | **7** | 100 | 0 | **8** | 6 | 0 |
| 19 | 12 | **30** | Presence of protocol for patients already taking opioids that includes pain reviews, lowest effective dose, avoidance of escalation of opioids postoperatively and use of non-opioid analgesia first-line. | Post Op | Pt or Pr Specific Px or DePx | Process | **8** | 100 | 0 | **7** | 100 | 0 | **8** | 6 | 0 |
| 19 | 35 | **51** | Opioids not prescribed for more than 3-7 days and do not include modified-release formulations. | Discharge | Pt or Pr Specific Px or DePx | Process | **8** | 100 | 0 | **7** | 100 | 0 | **7** | 6 | 0 |
| 19 | 65 | **25** | Patient given educational materials that emphasise the need and benefit of non-opioid non-pharmacological approaches to analgesia | Post Op | Patient Education | Process | **8** | 100 | 0 | **7** | 100 | 0 | **6** | 6 | 0 |
| 24 | 12 | **59** | Identify those at risk of ORADEs when prescribing opioids for use at home. Male, obese, over 65, greater comorbidities, pre-op opioid use, concurrent sedative medication use. | Discharge | ORADEs | Process | **8** | 100 | 0 | **7** | 90 | 10 | **8** | 6 | 0 |
| 24 | 35 | **5** | Identification of pre-operative use of opioid medications, including Daily Morphine Equivalent Dose. | Pre-Op | Preop Pt Optimisation | Process | **8** | 100 | 0 | **7** | 90 | 10 | **7** | 5 | 1 |
| 24 | 35 | **41** | Presence of a de-escalation plan for opioids prescribed on discharge, including a tapering plan for those taking >= 50mg MME for >= 3 weeks or no tapering if opioid naive and opioids are ceased once functional recovery is achieved. | Discharge | Pt or Pr Specific Px or DePx | Structural | **8** | 100 | 0 | **7** | 90 | 10 | **7** | 6 | 0 |
| 27 | 5 | **68** | Presence of plan or protocol if opioid abuse or misuse is detected. | Follow Up | Pt or Pr Specific Px or DePx | Structural | **8** | 100 | 0 | **7** | 80 | 20 | **9** | 6 | 0 |
| 27 | 12 | **36** | Patient given opioid specific discharge advice including not driving for up to 4 weeks until opioid dose is stable and managing post-operative pain. | Discharge | Patient Education | Process | **8** | 100 | 0 | **7** | 80 | 20 | **8** | 6 | 0 |
| 27 | 12 | **45** | Pain management plan and tapering strategies, including dose, amount supplied, and duration of 5 days and no longer than 7 days, clearly communicated to primary care team in a timely manner. | Discharge | Pt or Pr Specific Px or DePx | Process | **8** | 100 | 0 | **7** | 80 | 20 | **8** | 6 | 0 |
| 27 | 12 | **73** | Incidence of opioid related re-admissions. | Follow Up | Pt or Pr Specific Px or DePx | Outcome | **8** | 100 | 0 | **7** | 80 | 20 | **8** | 6 | 0 |
| 27 | 27 | **19** | A documented individualised intraoperative plan for patients already taking opioid medications, including use of regional, neuraxial techniques and non-opioid adjuncts as recommended by ANZCA FPM, with planned management of acute pain. | Intraop | Pt or Pr Specific Px or DePx | Process | **8** | 100 | 0 | **7** | 80 | 20 | **8** | 6 | 0 |
| 32 | 35 | **32** | Pharmacist or Pain team review of opiate prescribing if greater than 3 day use postoperatively. | Post Op | Pt or Pr Specific Px or DePx | Process | **8** | 90 | 10 | **7** | 90 | 10 | **7** | 6 | 0 |
| 33 | 27 | **37** | Patient education documented on pain management for mobilisation with deprescribing order: opioid, then NSAID, then paracetamol, with advice on drowsiness or worsening pain. | Discharge | Patient Education | Process | **8** | 100 | 0 | **6** | 90 | 10 | **8** | 6 | 0 |
| 34 | 12 | **67** | Hospital analgesic policies include strategies to support post-discharge assessment and follow-up of patients at risk of becoming chronic opioid users. | Follow Up | Pt or Pr Specific Px or DePx | Structural | **8** | 100 | 0 | **6** | 70 | 30 | **8** | 5 | 1 |
| 35 | 27 | **34** | Rate of ORADEs including severity and impact on length of stay. | Post Op | Pt or Pr Specific Px or DePx | Outcome | **8** | 90 | 10 | **6** | 90 | 10 | **8** | 6 | 0 |
| 36 | 27 | **62** | No repeat prescriptions of opioids following discharge, with new modified-release opioids only to be started in conjunction with specialist referral. | Follow Up | Pt or Pr Specific Px or DePx | Process | **8** | 90 | 10 | **6** | 80 | 20 | **8** | 6 | 0 |
| 37 | 27 | **42** | Electronic clinical quality measure (eCQM) to assess potentially inappropriate high dose postoperative opioid prescribing practices e.g an average daily dose ≥90 MME for the duration of postoperative opioid prescription in preoperatively opioid naïve patients. | Discharge | Pt or Pr Specific Px or DePx | Structural | **8** | 90 | 10 | **6** | 70 | 30 | **8** | 6 | 0 |
| 37 | 35 | **18** | Use of procedure-related PROSPECT recommendations for analgesia. | Intraop | Pt or Pr Specific Px or DePx | Process | **8** | 90 | 10 | **6** | 70 | 30 | **7** | 6 | 0 |
| 39 | 35 | **22** | Adherence to recovery/immediate postoperative opioid-sparing protocol, including continuing multimodal analgesia, avoiding opioid boluses, and simple analgesics. | Recovery | Pt or Pr Specific Px or DePx | Process | **8** | 90 | 10 | **6** | 60 | 40 | **7** | 6 | 0 |
| 40 | 12 | **65** | Reduction of unused opioid in the community. | Follow Up | Pt or Pr Specific Px or DePx | Outcome | **8** | 90 | 10 | **5** | 60 | 40 | **8** | 6 | 0 |
| 40 | 56 | **6** | Referral to specialist pain service for opioid weaning and optimisation and perioperative analgesic planning in patients with potential opioid tolerance or complex pain needs. | Pre-Op | Preop Pt Optimisation | Process | **8** | 90 | 10 | **5** | 60 | 40 | **6** | 6 | 0 |
| 42 | 35 | **40** | Presence of a patient group specific protocol for discharge opioid prescribing that includes calculating dose for discharge based upon past 24-hour use of opioids, recommends using lowest dose of opioids possible for the shortest duration, opioids and non-opioids to be prescribed separately. | Discharge | Pt or Pr Specific Px or DePx | Structural | **7** | 100 | 0 | **7** | 90 | 10 | **7** | 6 | 0 |
| 42 | 56 | **10** | Screening tool used to identify preoperatively patients at greater risk of postoperative Opioid Related Adverse Drug Events (ORADE’s). | Pre-Op | Preop Pt Optimisation | Process | **7** | 100 | 0 | **7** | 90 | 10 | **6** | 6 | 0 |
| 44 | 52 | **38** | Patient given BPS leaflet on managing post-operative pain. | Discharge | Patient Education | Process | **7** | 100 | 0 | **7** | 80 | 20 | **7** | 5 | 1 |
| 45 | 35 | **2** | Patient informed of risks of opioid medication, that post-op opioids will be a short course and deprescribed. | Pre-Op | Patient Education | Process | **7** | 90 | 10 | **7** | 100 | 0 | **7** | 6 | 0 |
| 46 | 35 | **48** | Frequency of slow-release opioids prescribed on discharge. | Discharge | Pt or Pr Specific Px or DePx | Process | **7** | 90 | 10 | **7** | 90 | 10 | **7** | 6 | 0 |
| 47 | 35 | **46** | Opioid present on hospital discharge prescription. | Discharge | Pt or Pr Specific Px or DePx | Process | **7** | 90 | 10 | **7** | 80 | 20 | **7** | 6 | 0 |
| 47 | 35 | **50** | Non-opioid adjuvant analgesia present on discharge prescription. | Discharge | Pt or Pr Specific Px or DePx | Process | **7** | 90 | 10 | **7** | 80 | 20 | **7** | 6 | 0 |
| 47 | 52 | **49** | Frequency of immediate-release opioids prescribed on discharge. | Discharge | Pt or Pr Specific Px or DePx | Process | **7** | 90 | 10 | **7** | 80 | 20 | **7** | 6 | 0 |
| 50 | 56 | **43** | Procedure specific post op prescribing guidelines to provide enough doses to cover 75% of patients. | Discharge | Pt or Pr Specific Px or DePx | Structural | **7** | 90 | 10 | **7** | 70 | 30 | **6** | 6 | 0 |
| 51 | 65 | **8** | Opioid Risk Tool (ORT) used to identify preoperatively patients at greater risk of persistent postoperative opioid use (PPOU). | Pre-Op | Preop Pt Optimisation | Process | **7** | 100 | 0 | **6** | 100 | 0 | **6** | 5 | 1 |
| 52 | 35 | **13** | Documentation of ‘universal precautions’ when initiating perioperative opioids. | Pre-Op | Pt or Pr Specific Px or DePx | Process | **7** | 90 | 10 | **6** | 90 | 10 | **7** | 6 | 0 |
| 53 | 35 | **71** | PPOU: ongoing or increased (relative to pre-op) opioid use at 90-180/365 days post discharge. | Follow Up | Pt or Pr Specific Px or DePx | Outcome | **7** | 90 | 10 | **6** | 80 | 20 | **7** | 6 | 0 |
| 54 | 35 | **72** | Time to opioid cessation: a period without an opioid prescription equivalent to three times the estimated supply duration in preoperatively opioid naïve patients. | Follow Up | Pt or Pr Specific Px or DePx | Outcome | **7** | 90 | 10 | **6** | 70 | 30 | **7** | 6 | 0 |
| 55 | 27 | **70** | Use of higher dosage of opioids at any time (>50-60 MME). | Follow Up | Pt or Pr Specific Px or DePx | Outcome | **7** | 80 | 20 | **6** | 80 | 20 | **8** | 6 | 0 |
| 56 | 35 | **15** | Individualised perioperative pain management plan communicated to surgical, anaesthetic, care of elderly and frailty teams. | Pre-Op | Pt or Pr Specific Px or DePx | Process | **7** | 100 | 0 | **5** | 60 | 40 | **7** | 6 | 0 |
| 57 | 56 | **14** | Opioid medications not increased preoperatively. | Pre-Op | Pt or Pr Specific Px or DePx | Process | **7** | 100 | 0 | **5** | 50 | 50 | **6** | 6 | 0 |
| 58 | 70 | **31** | Maximum daily oral MME for an opioid-naïve patient of 50mg. | Post Op | Pt or Pr Specific Px or DePx | Process | **7** | 90 | 10 | **5** | 80 | 20 | **5** | 4 | 2 |
| 59 | 56 | **44** | Procedure specific prescribing limits built into electronic patient record. | Discharge | Pt or Pr Specific Px or DePx | Structural | **7** | 90 | 10 | **5** | 70 | 30 | **6** | 5 | 1 |
| 60 | 35 | **53** | Senior review of need for discharge opioid medication. | Discharge | Pt or Pr Specific Px or DePx | Process | **7** | 80 | 20 | **5** | 50 | 50 | **7** | 6 | 0 |
| 61 | 65 | **52** | Patients should not be discharged on strong opioids. | Discharge | Pt or Pr Specific Px or DePx | Process | **7** | 60 | 40 | **5** | 60 | 40 | **6** | 4 | 2 |
| *62* | *27* | ***7*** | *Referral for counselling or psychosocial support for patients with complex pain needs.* | *Pre-Op* | *Preop Pt Optimisation* | *Process* | ***7*** | *90* | *10* | ***4*** | *30* | *70* | ***8*** | *6* | *0* |
| *63* | *27* | ***60*** | *Prescribers sent quarterly reports on their prescribing compared to guidelines.* | *Follow Up* | *Staff Education* | *Structural* | ***7*** | *90* | *10* | ***3*** | *40* | *60* | ***8*** | *4* | *2* |
| 64 | 52 | **54** | Presence of recording tool for opioids used during inpatient stay. | Discharge | Pt or Pr Specific Px or DePx | Process | **6** | 100 | 0 | **6** | 80 | 20 | **7** | 6 | 0 |
| 64 | 56 | **56** | Total milligram of morphine equivalents (MME) consumed during 24h prior to discharge. | Discharge | Pt or Pr Specific Px or DePx | Outcome | **6** | 100 | 0 | **6** | 80 | 20 | **6** | 6 | 0 |
| 66 | 56 | **47** | Procedure-specific mean discharge MME prescribed. | Discharge | Pt or Pr Specific Px or DePx | Process | **6** | 100 | 0 | **5** | 80 | 20 | **6** | 6 | 0 |
| 67 | 65 | **9** | Biopsychosocial assessment of pain and history of use of analgesic medications including opioids. | Pre-Op | Preop Pt Optimisation | Process | **6** | 100 | 0 | **5** | 70 | 30 | **6** | 6 | 0 |
| 68 | 52 | **61** | Presence of process to assess opioids prescribed versus opioids used following surgical procedures to allow tailoring of opioid prescriptions to need for a patient group/specific procedure. | Follow Up | Pt or Pr Specific Px or DePx | Structural | **6** | 100 | 0 | **5** | 50 | 50 | **7** | 5 | 1 |
| 69 | 56 | **20** | Patient reviewed in PACU for new risk factors for PPOU, including formation of a stoma. | Recovery | Pt or Pr Specific Px or DePx | Process | **6** | 90 | 10 | **5** | 70 | 30 | **6** | 6 | 0 |
| 70 | 65 | **57** | Total milligram of morphine equivalents (MME) consumed during hospital stay. | Discharge | Pt or Pr Specific Px or DePx | Outcome | **6** | 70 | 30 | **5** | 60 | 40 | **6** | 5 | 1 |
| *71* | *56* | ***66*** | *Post operative prescription considered to have been given if opioids dispensed between 2-7 days following discharge.* | *Follow Up* | *Pt or Pr Specific Px or DePx* | *Outcome* | ***6*** | *90* | *10* | ***4*** | *40* | *60* | ***6*** | *4* | *2* |
| *72* | *70* | ***11*** | *Wean preoperative opioids to target of 60mg Morphine Equivalent Dose or below and by no more than 10% per week.* | *Pre-Op* | *Preop Pt Optimisation* | *Process* | ***6*** | *80* | *20* | ***4*** | *20* | *80* | ***5*** | *5* | *1* |
| 73 | 70 | **58** | Procedure specific mean daily inpatient MME used. | Discharge | Pt or Pr Specific Px or DePx | Outcome | **5** | 80 | 20 | **5** | 60 | 40 | **5** | 5 | 1 |
