# Supplementary material for: Development of actionable quality indicators and an implementation toolkit for perioperative opioid stewardship in colorectal cancer in the UK Yorkshire and Humber region: a modified RAND consensus study
Source: BMJ Open. 2025 Sep 30;15(9):e092675. doi: 10.1136/bmjopen-2024-092675 (PMC12506214; doi:10.1136/bmjopen-2024-092675)
Supplement: online supplemental file 5 [file bmjopen-15-9-s005.docx]

Supplemental Table 1 – Final Indicator Scoring

| **Quality Indicator Number** | **Quality Indicator** | **Operation pathway stage** | **Round 2: Online Survey** | | **Round 3: Patient Panel** | **Round 4: Consensus Meeting** | | | | | **Result** |
| --- | --- | --- | --- | --- | --- | --- | --- | --- | --- | --- | --- |
|  |  |  | **Individual expert rating** |  | **Individual patient rating** | **Group discussion** | **Individual expert and PPI represent-ative rating** |  |  |  |  |
|  |  |  | **Relevance (median score)** | **Action-ability (median score)** | **Import-ance (median score)** |  | **Relevance (median score)** | **Action-ability (median score)** | **Feasibility (median score)** | **Validity (median score)** |  |
| 1 | Discussion with patient regarding realistic expectations of pain post-op and pain management | Pre-operative | 8 | 8 | 8 | Rephrased | 9 | 8 | 5 | 7 | Rejected |
| 2 | Patient informed of risks of opioid medication, that post-op opioids will be a short course and deprescribed | Pre-operative | 7 | 7 | 7 | Merged with 1 |  |  |  |  |  |
| 3 | Patient provided educational materials on pain, including a documented pain management plan | Pre-operative | 8 | 8 | 5 | Rejected after discussion |  |  |  |  |  |
| 4 | Ongoing multi-professional education for staff on opioid stewardship and need for multi-modal analgesia throughout the patient pathway | Pre-operative | 9 | 8 | 9 | Rephrased | 8 | 8 | 7 | 8 | Rejected |
| 5 | Identification of pre-operative use of opioid medications, including Daily Morphine Equivalent Dose | Pre-operative | 8 | 7 | 7 | Rejected after discussion |  |  |  |  |  |
| 6 | Referral to specialist pain service for opioid weaning and optimisation and perioperative analgesic planning in patients with potential opioid tolerance or complex pain needs | Pre-operative | 8 | 5 | 6 | Rejected after discussion |  |  |  |  |  |
| 7 | Referral for counselling or psychosocial support for patients with complex pain needs | Pre-operative | 7 | 4 | 8 | Not discussed |  |  |  |  |  |
| 8 | Opioid Risk Tool (ORT) used to identify preoperatively patients at greater risk of persistent postoperative opioid use (PPOU) | Pre-operative | 7 | 6 | 6 | Rejected after discussion |  |  |  |  |  |
| 9 | Biopsychosocial assessment of pain and history of use of analgesic medications including opioids | Pre-operative | 6 | 5 | 6 | Rejected after discussion |  |  |  |  |  |
| 10 | Screening tool used to identify preoperatively patients at greater risk of postoperative Opioid Related Adverse Drug Events (ORADE’s) | Pre-operative | 7 | 7 | 6 | Merged with 12 |  |  |  |  |  |
| 11 | Wean preoperative opioids to target of 60mg Morphine Equivalent Dose or below and by no more than 10% per week | Pre-operative | 6 | 4 | 5 | Not discussed |  |  |  |  |  |
| **12** | **Presence of a protocol to reduce perioperative opioid use with preoperative multimodal analgesia, including adjuncts such as NSAID’s and medications that may limit pain experience such as antiemetics** | **Pre-operative** | **8** | **8** | **7** | **Rephrased** | **9** | **8** | **7** | **8** | **Accepted** |
| 13 | Documentation of ‘universal precautions’ when initiating perioperative opioids | Pre-operative | 7 | 6 | 7 | Rejected after discussion |  |  |  |  |  |
| 14 | Opioid medications are not increased preoperatively | Pre-operative | 7 | 5 | 6 | Rejected after discussion |  |  |  |  |  |
| 15 | Individualised perioperative pain management plan communicated to surgical, anaesthetic, care of elderly and frailty teams | Pre-operative | 7 | 5 | 7 | Merged with 12 |  |  |  |  |  |
| 16 | Presence of an opioid-sparing protocol for intra operative use which includes minimally invasive surgery, regional blocks, neuraxial techniques and multimodal analgesia | Intra-operative | 9 | 8 | 9 | Merged with 12 |  |  |  |  |  |
| 17 | Adherence to intraoperative opioid-sparing protocol with documented use of multimodal approach which includes minimally invasive surgery, regional blocks, neuraxial techniques, non-pharmacological and multimodal analgesia | Intra-operative | 8 | 8 | 8 | Rejected after discussion |  |  |  |  |  |
| 18 | Use of procedure-related PROSPECT recommendations for analgesia | Intra-operative | 8 | 6 | 7 | Rejected after discussion |  |  |  |  |  |
| 19 | A documented individualised intraoperative plan for patients already taking opioid medications, including use of regional, neuraxial techniques and non-opioid adjuncts as recommended by ANZCA FPM, with planned management of acute pain | Intra-operative | 8 | 7 | 8 | Rejected after discussion |  |  |  |  |  |
| 20 | Patient reviewed in PACU for new risk factors for PPOU, including formation of a stoma | Recovery | 6 | 5 | 6 | Rejected after discussion |  |  |  |  |  |
| 21 | Presence of an opioid-sparing protocol for recovery/immediate postoperative use which includes regional blocks, non-pharmacological treatments, standardized rescue, multimodal analgesia and avoidance of PCA and PCEAs if able to take oral fluids and analgesia | Recovery | 9 | 7 | 8 | Merged with 12 |  |  |  |  |  |
| 22 | Adherence to recovery/immediate postoperative opioid-sparing protocol, including continuing multimodal analgesia, avoiding opioid boluses, and simple analgesics | Recovery | 8 | 6 | 7 | Rejected after discussion |  |  |  |  |  |
| **23** | **Regular post-operative functional assessment of pain which includes ability to cough and deep breathe** | **Recovery** | **8** | **8** | **9** | **Rephrased** | **8** | **8** | **8** | **8** | **Accepted** |
| 24 | Assessment of sedation in PACU | Recovery | 8 | 8 | 8 | Rejected after discussion |  |  |  |  |  |
| 25 | Patient given educational materials that emphasise the need and benefit of non-opioid non-pharmacological approaches to analgesia | Post-operative | 8 | 7 | 6 | Rejected after discussion |  |  |  |  |  |
| 26 | Presence of a referral route to an acute pain service that includes pathways for opioid tolerant patients and readmissions due to pain or opioids | Post-operative | 8 | 8 | 8 | Merged with 12 |  |  |  |  |  |
| 27 | Daily postoperative pain review | Post-operative | 9 | 7 | 9 | Merged with 12 |  |  |  |  |  |
| 28 | Presence of a postoperative opioid-sparing protocol which includes regional blocks, non-pharmacological approaches, simple analgesia and multimodal analgesia, with oral medications prioritised, particularly those that can be administered by one nurse (oramorph rather than oxycodone), avoidance of long-acting and im opioids and prescribed according to renal function and age | Post-operative | 8 | 7 | 8 | Merged with 12 |  |  |  |  |  |
| 29 | Adherence to a postoperative opioid-sparing protocol | Post-operative | 8 | 7 | 8 | Merged with 12 |  |  |  |  |  |
| 30 | Presence of protocol for patients already taking opioids that includes pain reviews, lowest effective dose, avoidance of escalation of opioids postoperatively and use of non-opioid analgesia first-line | Post-operative | 8 | 7 | 8 | Merged with 12 |  |  |  |  |  |
| 31 | Maximum daily oral MME for an opioid-naïve patient of 50mg | Post-operative | 7 | 5 | 5 | Rejected after discussion |  |  |  |  |  |
| 32 | Pharmacist or Pain team review of opiate prescribing if greater than 3 days use postoperatively | Post-operative | 8 | 7 | 7 | Merged with 12 |  |  |  |  |  |
| 33 | Patients receiving postoperative opioids have sedation score documented | Post-operative | 9 | 8 | 9 | Merged with 23 |  |  |  |  |  |
| 34 | Rate of ORADEs including severity and impact on length of stay | Post-operative | 8 | 6 | 8 | Rejected after discussion |  |  |  |  |  |
| **35** | **Patient given education leaflet on safe administration, storage, weaning and disposal of unused opioids and avoidance of opioid diversion** | **Discharge** | **8** | **8** | **9** | **Rephrased** | **8** | **8** | **8** | **8** | **Accepted** |
| 36 | Patient given opioid specific discharge advice including not driving for up to 4 weeks until opioid dose is stable and managing post-operative pain | Discharge | 8 | 7 | 8 | Merged with 35 |  |  |  |  |  |
| 37 | Patient education documented on pain management for mobilisation with deprescribing order: opioid, then NSAID, then paracetamol, with advice on drowsiness or worsening pain | Discharge | 8 | 6 | 8 | Merged with 35 |  |  |  |  |  |
| 38 | Patient given BPS leaflet on managing post-operative pain | Discharge | 7 | 7 | 7 | Merged with 35 |  |  |  |  |  |
| 39 | Patient given point of contact for ongoing pain issue | Discharge | 9 | 8 | 9 | Merged with 35 |  |  |  |  |  |
| 40 | Presence of a patient group specific protocol for discharge opioid prescribing that includes calculating dose for discharge based upon past 24-hour use of opioids, recommends using lowest dose of opioids possible for the shortest duration, opioids and non-opioids to be prescribed separately | Discharge | 7 | 7 | 7 | Merged with 12 |  |  |  |  |  |
| 41 | Presence of a de-escalation plan for opioids prescribed on discharge, including a tapering plan for those taking >= 50mg MME for >= 3 weeks or no tapering if opioid naive and opioids are ceased once functional recovery is achieved | Discharge | 8 | 7 | 7 | Rejected after discussion |  |  |  |  |  |
| 42 | Electronic clinical quality measure (eCQM) to assess potentially inappropriate high dose postoperative opioid prescribing practices e.g an average daily dose ≥90 MME for the duration of postoperative opioid prescription in preoperatively opioid naïve patients | Discharge | 8 | 6 | 8 | Rejected after discussion |  |  |  |  |  |
| 43 | Procedure specific post op prescribing guidelines to provide enough doses to cover 75% of patients | Discharge | 7 | 7 | 6 | Rejected after discussion |  |  |  |  |  |
| 44 | Procedure specific prescribing limits built into electronic patient record | Discharge | 7 | 5 | 6 | Rejected after discussion |  |  |  |  |  |
| 45 | Pain management plan and tapering strategies, including dose, amount supplied, and duration of 5 days and no longer than 7 days, clearly communicated to primary care team in a timely manner | Discharge | 8 | 7 | 8 | Rejected after discussion |  |  |  |  |  |
| **46** | **Patients should not be discharged on strong opioids (BNF definition) unless reviewed by senior clinician before discharge or taking for another condition pre-surgery** | **Discharge** | **7** | **7** | **7** | **Rephrased** | **8** | **8** | **7** | **8** | **Accepted** |
| 47 | Procedure-specific mean discharge MME prescribed | Discharge | 6 | 5 | 6 | Merged with 46 |  |  |  |  |  |
| 48 | Frequency of slow-release opioids prescribed on discharge | Discharge | 7 | 7 | 7 | Merged with 46 |  |  |  |  |  |
| 49 | Frequency of immediate-release opioids prescribed on discharge | Discharge | 7 | 7 |  | Merged with 46 |  |  |  |  |  |
| 50 | Non-opioid adjuvant analgesia present on discharge prescription | Discharge | 7 | 7 | 7 | Merged with 46 |  |  |  |  |  |
| 51 | Opioids not prescribed for more than 3-7 days and do not include modified-release formulations | Discharge | 8 | 7 | 7 | Merged with 46 |  |  |  |  |  |
| 52 | Patients should not be discharged on strong opioids | Discharge | 7 | 5 | 6 | Merged with 46 |  |  |  |  |  |
| 53 | Senior review of need for discharge opioid medication | Discharge | 7 | 5 | 7 | Merged with 46 |  |  |  |  |  |
| 54 | Presence of recording tool for opioids used during inpatient stay | Discharge | 6 | 6 | 7 | Rejected after discussion |  |  |  |  |  |
| 55 | Use of ‘reverse pain ladder’ for de-escalation of opioids | Discharge | 8 | 8 | 8 | Rejected after discussion |  |  |  |  |  |
| 56 | Total milligram of morphine equivalents (MME) consumed during 24h prior to discharge | Discharge | 6 | 6 | 6 | Rejected after discussion |  |  |  |  |  |
| 57 | Total milligram of morphine equivalents (MME) consumed during hospital stay | Discharge | 6 | 5 | 6 | Rejected after discussion |  |  |  |  |  |
| 58 | Procedure specific mean daily inpatient MME used | Discharge | 5 | 5 | 5 | Rejected after discussion |  |  |  |  |  |
| 59 | Identify those at risk of ORADEs when prescribing opioids for use at home. Male, obese, over 65, greater comorbidities, pre-op opioid use, concurrent sedative medication use | Discharge | 8 | 7 | 8 | Merged with 12 |  |  |  |  |  |
| 60 | Prescribers sent quarterly reports on their prescribing compared to guidelines | Discharge | 7 | 3 | 8 | Not discussed |  |  |  |  |  |
| 61 | Presence of process to assess opioids prescribed versus opioids used following surgical procedures to allow tailoring of opioid prescriptions to need for a patient group/specific procedure | Follow Up | 6 | 5 | 7 | Rejected after discussion |  |  |  |  |  |
| 62 | No repeat prescriptions of opioids following discharge, with new modified-release opioids only to be started in conjunction with specialist referral | Follow Up | 8 | 6 | 8 | Rejected after discussion |  |  |  |  |  |
| 63 | A patient still on opioids at 90 days to have review in primary or secondary care or pain service referral | Follow Up | 9 | 7 | 9 | Rejected after discussion |  |  |  |  |  |
| 64 | Screening to identify patients who were opioid-naive pre-operatively still taking opioids at first review | Follow Up | 9 | 6 | 9 | Rephrased | 8 | 7 | 6 | 7 | Rejected |
| 65 | Reduction of unused opioid in the community | Follow Up | 8 | 5 | 8 | Rejected after discussion |  |  |  |  |  |
| 66 | Post operative prescription considered to have been given if opioids dispensed between 2-7 days following discharge | Follow Up | 6 | 4 | 6 | Not discussed |  |  |  |  |  |
| 67 | Hospital analgesic policies include strategies to support post-discharge assessment and follow-up of patients at risk of becoming chronic opioid users | Follow Up | 8 | 6 | 8 | Rejected after discussion |  |  |  |  |  |
| 68 | Presence of plan or protocol if opioid abuse or misuse is detected | Follow Up | 8 | 7 | 9 | Rejected after discussion |  |  |  |  |  |
| 69 | Procedure for return and disposal of unused opioids | Follow Up | 9 | 7 | 9 | Merged with 35 |  |  |  |  |  |
| 70 | Use of higher dosage of opioids at any time (>50-60 MME) | Follow Up | 7 | 6 | 8 | Rejected after discussion |  |  |  |  |  |
| 71 | PPOU: ongoing or increased (relative to pre-op) opioid use at 90-180/365 days post discharge | Follow Up | 7 | 6 | 7 | Rejected after discussion |  |  |  |  |  |
| 72 | Time to opioid cessation: a period without an opioid prescription equivalent to three times the estimated supply duration in preoperatively opioid naïve patients | Follow Up | 7 | 6 | 7 | Rejected after discussion |  |  |  |  |  |
| 73 | Incidence of opioid related re-admissions | Follow Up | 8 | 7 | 8 | Rejected after discussion |  |  |  |  |  |
