# Supplementary material for: Development of actionable quality indicators and an implementation toolkit for perioperative opioid stewardship in colorectal cancer in the UK Yorkshire and Humber region: a modified RAND consensus study
Source: BMJ Open. 2025 Sep 30;15(9):e092675. doi: 10.1136/bmjopen-2024-092675 (PMC12506214; doi:10.1136/bmjopen-2024-092675)
Supplement: online supplemental file 6 [file bmjopen-15-9-s006.docx]

Supplemental table 2 – ERIC strategies for indicators

| ERIC Strategies | Indicator 1 - Opioid Stewardship Protocol | Indicator 2 - Post-Operative Pain Assessment | Indicator 3 - Patient Discharge Education & Leaflet | Indicator 4 - Discharge Review for Strong Opioids |
| --- | --- | --- | --- | --- |
| Access new funding | 179% | 149% | 215% | 258% |
| Alter incentive/allowance structures | 391% | 157% | 298% | 485% |
| Alter patient/consumer fees | 55% | 49% | 68% | 75% |
| Assess for readiness and identify barriers and facilitators | 510% | 160% | 364% | 633% |
| Audit and provide feedback | 132% | 46% | 148% | 239% |
| Build a coalition | 273% | 100% | 232% | 373% |
| Capture and share local knowledge | 224% | 134% | 223% | 323% |
| Centralize technical assistance | 34% | 24% | 52% | 56% |
| Change accreditation or membership reqs | 26% | 12% | 27% | 38% |
| Change liability laws | 27% | 8% | 7% | 34% |
| Change physical structure and equipment | 98% | 55% | 90% | 113% |
| Change record system | 37% | 11% | 48% | 48% |
| Change service sites | 32% | 18% | 32% | 35% |
| Conduct cyclical small tests of change | 162% | 90% | 163% | 252% |
| Conduct educational meetings | 231% | 174% | 185% | 278% |
| Conduct educational outreach visits | 133% | 90% | 149% | 173% |
| Conduct local consensus discussions | 353% | 116% | 276% | 498% |
| Conduct local needs assessment | 365% | 100% | 266% | 419% |
| Conduct ongoing training | 82% | 99% | 151% | 129% |
| Create a learning collaborative | 227% | 138% | 213% | 328% |
| Create new clinical teams | 48% | 15% | 44% | 49% |
| Create or change credentialing and/or licensure standards | 61% | 35% | 49% | 88% |
| Develop a formal implementation blueprint | 187% | 139% | 302% | 302% |
| Develop academic partnerships | 57% | 42% | 90% | 75% |
| Develop an implementation glossary | 20% | 14% | 40% | 28% |
| Develop and implement tools for quality monitoring | 134% | 42% | 189% | 222% |
| Develop and organize quality monitoring systems | 73% | 22% | 111% | 147% |
| Develop disincentives | 46% | 15% | 69% | 81% |
| Develop educational materials | 121% | 147% | 136% | 132% |
| Develop resource sharing agreements | 61% | 45% | 93% | 105% |
| Distribute educational materials | 100% | 100% | 68% | 109% |
| Facilitate relay of clinical data to providers | 109% | 45% | 113% | 154% |
| Facilitation | 213% | 99% | 230% | 332% |
| Fund and contract for clinical innovation | 156% | 85% | 155% | 206% |
| Identify and prepare champions | 521% | 228% | 420% | 746% |
| Identify early adopters | 233% | 104% | 206% | 378% |
| Increase demand | 186% | 81% | 171% | 184% |
| Inform local opinion leaders | 275% | 112% | 219% | 415% |
| Intervene with patients/consumers to enhance uptake & adherence | 110% | 61% | 121% | 121% |
| Involve executive boards | 198% | 58% | 210% | 258% |
| Involve patients/consumers and family members | 268% | 88% | 222% | 267% |
| Make billing easier | 41% | 37% | 69% | 77% |
| Make training dynamic | 40% | 48% | 87% | 83% |
| Mandate change | 120% | 59% | 94% | 147% |
| Model and simulate change | 110% | 42% | 125% | 179% |
| Obtain and use patients/consumers and family feedback | 205% | 65% | 203% | 201% |
| Obtain formal commitments | 141% | 73% | 143% | 155% |
| Organize clinician implementation team meetings | 136% | 80% | 169% | 235% |
| Place innovation on fee for service lists/formularies | 47% | 33% | 80% | 86% |
| Prepare patients/consumers to be active participants | 156% | 59% | 128% | 119% |
| Promote adaptability | 211% | 94% | 197% | 357% |
| Promote network weaving | 131% | 64% | 116% | 164% |
| Provide clinical supervision | 28% | 36% | 30% | 45% |
| Provide local technical assistance | 65% | 44% | 152% | 144% |
| Provide ongoing consultation | 142% | 81% | 157% | 194% |
| Purposely reexamine the implementation | 114% | 36% | 159% | 203% |
| Recruit, designate and train for leadership | 211% | 98% | 159% | 289% |
| Remind clinicians | 7% | 7% | 19% | 15% |
| Revise professional roles | 75% | 46% | 67% | 97% |
| Shadow other experts | 41% | 42% | 41% | 68% |
| Stage implementation scale up | 120% | 85% | 136% | 153% |
| Start a dissemination organization | 32% | 4% | 32% | 34% |
| Tailor strategies | 210% | 87% | 165% | 311% |
| Use advisory boards and workgroups | 224% | 77% | 193% | 232% |
| Use an implementation adviser | 130% | 82% | 180% | 182% |
| Use capitated payments | 16% | 16% | 37% | 36% |
| Use data experts | 53% | 18% | 69% | 104% |
| Use data warehousing techniques | 9% | 0% | 21% | 27% |
| Use mass media | 89% | 60% | 73% | 92% |
| Use other payment schemes | 54% | 54% | 53% | 74% |
| Use train the trainer strategies | 72% | 57% | 74% | 91% |
| Visit other sites | 137% | 56% | 116% | 205% |
| Work with educational institutions | 43% | 23% | 32% | 54% |
