# Supplementary material for: Development of actionable quality indicators and an implementation toolkit for perioperative opioid stewardship in colorectal cancer in the UK Yorkshire and Humber region: a modified RAND consensus study
Source: BMJ Open. 2025 Sep 30;15(9):e092675. doi: 10.1136/bmjopen-2024-092675 (PMC12506214; doi:10.1136/bmjopen-2024-092675)
Supplement: online supplemental file 7 [file bmjopen-15-9-s007.docx]

| ERIC Improvement Strategy identified | Indicators | Toolkit contents to support |
| --- | --- | --- |
| Access new funding | 1-2-3-4 | Funding for Yorkshire registry of patient leaflets.  Print resources and/or online materials.  Provide statements of evidence base for indicators and standards for Trust executives to mobilise resources. |
| Alter incentive/allowance structures | 1-2-3-4 | No financial incentive source identified.  Promote within AXA accreditation standard achievements.  Evidence of application of indicators into practice can be used to support achievement in quality improvement schemes. |
| Alter patient/consumer fees | 1 | Not applicable in UK. |
| Assess for readiness and identify barriers and facilitators | 1-2-3-4 | BCIP team to review barriers and facilitators with local team. Provide customisable action plans on how to operationalise indicators. |
| Audit and provide feedback | 1-3-4 | BCIP audit tool and feedback reports developed, including inclusion and exclusion criteria for denominators, evidence base, reaudit cycle. |
| Build a coalition | 1-2-3-4 | BCIP opioid stewardship quality improvement collaborative established with scheduled regular online meetings and educational updates. |
| Capture and share local knowledge | 1-2-3-4 | BCIP opioid stewardship quality improvement collaborative established with scheduled regular online meetings and educational updates. |
| Change physical structure and equipment | 1-2-3-4 | BCIP repository of patient information leaflets and adaptable opioid stewardship protocol.  Information on BCIP website regarding repository.  Information for staff noticeboards on opioid stewardship and recommendations. |
| Conduct cyclical small tests of change | 1-2-3-4 | Advice on implementing change, including template for plan, do, study, act (PDSA) cycles. |
| Conduct educational meetings | 1-2-3-4 | Schedule of BCIP educational meetings (including Toolkit launch event, lunch and learn events). |
| Conduct educational outreach visits | 1-2-3-4 | BCIP visits to Trusts for educational outreach, including local needs assessment, facilitation of action plan development. |
| Conduct local consensus discussions | 1-2-3-4 | BCIP opioid stewardship quality improvement collaborative established with scheduled regular online meetings and educational updates. |
| Conduct local needs assessment | 1-2-3-4 | BCIP visits to Trusts for educational outreach, including local needs assessment. |
| Conduct ongoing training | 1-2-3-4 | Schedule of BCIP educational meetings (including Toolkit launch event, lunch and learn events). |
| Create a learning collaborative | 1-2-3-4 | BCIP opioid stewardship quality improvement collaborative established with scheduled regular online meetings and educational updates. |
| Create or change credentialing and/or licensure standards | 1-4 | Information regarding how to use indicator attainment and improvements in documentation for assessments and Care Quality Commission reviews. |
| Develop a formal implementation blueprint | 1-2-3-4 | BCIP action plan template. |
| Develop academic partnerships | 1-3 | Information on BCIP and contact details. |
| Develop and implement tools for quality monitoring | 1-3-4 | BCIP audit tool and feedback reports developed, including inclusion and exclusion criteria for denominators, evidence base, reaudit cycle. |
| Develop and organize quality monitoring systems | 1-3-4 | BCIP audit tool and feedback reports developed, including inclusion and exclusion criteria for denominators, evidence base, reaudit cycle. |
| Develop disincentives | 3 | Information from BCIP patient and public group on importance of patient information leaflet to patients. |
| Develop educational materials | 1-2-3-4 | BCIP audit tool and feedback reports developed, including inclusion and exclusion criteria for denominators, evidence base, reaudit cycle. |
| Develop resource sharing agreements | 1-3-4 | BCIP repository of patient information leaflets and adaptable opioid stewardship protocol. |
| Distribute educational materials | 1-2-4 | BCIP repository of patient information leaflets and adaptable opioid stewardship protocol. |
| Facilitate relay of clinical data to providers | 1-3-4 | BCIP annual MDT reports include reminder of opioid stewardship and toolkit availability. |
| Facilitation | 1-2-3-4 | Signpost to audit and service improvement teams within Trusts.  BCIP opioid stewardship quality improvement collaborative established with scheduled regular online meetings and educational updates. |
| Fund and contract for clinical innovation | 1-2-3-4 | Information for commissioners and managers regarding BCIP opioid stewardship initiatives.  Informatsiion regarding regional peri-operative groups e.g. WYAT. |
| Identify and prepare champions | 1-2-3-4 | Local champion identified in establishing BCIP opioid stewardship quality improvement collaboratives. |
| Identify early adopters | 1-2-3-4 | Case studies from positive deviants with information regarding how change was achieved. |
| Increase demand | 1-2-3-4 | Audit and feedback.  Patient posters with information regarding importance of opioid stewardship for wards, printed by YCR.  Signposts on engaging with management. |
| Inform local opinion leaders | 1-2-3-4 | Local champion identified in establishing BCIP opioid stewardship quality improvement collaboratives.  Ongoing BCIP involvement in improving opioid stewardship. |
| Intervene with patients/consumers to enhance uptake & adherence | 1-2-3-4 | Patient posters with information regarding importance of opioid stewardship for wards, printed by YCR. |
| Involve executive boards | 1-2-3-4 | Information for commissioners and managers regarding BCIP opioid stewardship initiatives. |
| Involve patients/consumers and family members | 1-2-3-4 | Patient posters with information regarding importance of opioid stewardship for wards, printed by YCR. |
| Make billing easier | 3 | Not applicable in UK. |
| Make training dynamic | 3 | BCIP opioid stewardship quality improvement collaborative established with scheduled regular online meetings and educational updates. |
| Mandate change | 1-2-3-4 | BCIP audit tool and feedback reports developed, including inclusion and exclusion criteria for denominators, evidence base, reaudit cycle. |
| Model and simulate change | 1-3-4 | BCIP to lead and provide positive face of opioid stewardship.  Case studies from positive deviants with information regarding how change was achieved. |
| Obtain and use patients/consumers and family feedback | 1-2-3-4 | Patient feedback forms at local level. |
| Obtain formal commitments | 1-2-3-4 | BCIP opioid stewardship quality improvement collaborative established with formal commitment agreement to be signed by local identified champion. |
| Organize clinician implementation team meetings | 1-2-3-4 | BCIP visits to Trusts for educational outreach, including local needs assessment, facilitation of action plan development. |
| Place innovation on fee for service lists/formularies | 3-4 | Not applicable in UK. |
| Prepare patients/consumers to be active participants | 1-2-3-4 | Patient posters with information regarding importance of opioid stewardship for wards, printed by YCR.  Patient feedback forms at local level. |
| Promote adaptability | 1-2-3-4 | BCIP patient information leaflet and protocol for opioid stewardship documents for local tailoring.  Action plan developed with local leads. |
| Promote network weaving | 1-2-3-4 | BCIP opioid stewardship quality improvement collaborative established with scheduled regular online meetings and educational updates.  Schedule of BCIP educational meetings (including Toolkit launch event, lunch and learn events). |
| Provide local technical assistance | 1-3-4 | BCIP contact details for further support. |
| Provide ongoing consultation | 1-2-3-4 | Ongoing updates and provide new evidence for support for indicators by BCIP.  Recruit, designate and train local champion for leadership & encourage reflection. |
| Purposely reexamine the implementation | 1-3-4 | BCIP visits to Trusts for educational outreach, including local needs assessment, facilitation of action plan development and reviews.  Advice on implementing change, including template for plan, do, study, act (PDSA) cycles. |
| Recruit, designate and train for leadership | 1-2-3-4 | Recruit, designate and train local champion for leadership. |
| Revise professional roles | 1-4 | Recruit, designate and train local champion for leadership.  Information regarding senior clinician role in opioid review upon patient discharge. |
| Stage implementation scale up | 1-2-3-4 | BCIP visits to Trusts for educational outreach, including local needs assessment, facilitation of action plan development and reviews.  Advice on implementing change, including template for plan, do, study, act (PDSA) cycles.  Suggestion of starting in one area (for example, elective surgery patients) before adding additional areas (patients undergoing emergency surgery). |
| Tailor strategies | 1-2-3-4 | BCIP patient information leaflet and protocol for opioid stewardship documents for local tailoring.  Action plan developed with local leads. |
| Use advisory boards and workgroups | 1-2-3-4 | Central support from BCIP with contact details for lead clinicians.  BCIP opioid stewardship quality improvement collaborative established with scheduled regular online meetings and educational updates. |
| Use an implementation adviser | 1-2-3-4 | Contact details for BCIP and local implementation lead.  Signpost to audit and service improvement teams within Trusts. |
| Use data experts | 1-3-4 | Contact details for BCIP data specialist and programme manager. |
| Use mass media | 1-2-3-4 | Information on BCIP website regarding repository.  Social media links (to BCIP and YCR feeds). |
| Use other payment schemes | 1-2 | Not applicable in UK. |
| Use train the trainer strategies | 1-2-3-4 | Central support from BCIP with contact details for lead clinicians.  Recruit, designate and train local champion for leadership. |
| Visit other sites | 1-2-3-4 | BCIP opioid stewardship quality improvement collaborative established with scheduled regular online meetings and educational updates.  Case studies with contact details from positive deviants with information regarding how change was achieved. |
