# Supplementary material for: Development of actionable quality indicators and an implementation toolkit for perioperative opioid stewardship in colorectal cancer in the UK Yorkshire and Humber region: a modified RAND consensus study
Source: BMJ Open. 2025 Sep 30;15(9):e092675. doi: 10.1136/bmjopen-2024-092675 (PMC12506214; doi:10.1136/bmjopen-2024-092675)
Supplement: Supplementary file 8 [file bmjopen-2024-092675.reviewer_comments.pdf]

# PEER REVIEW HISTORY

BMJ Open publishes all reviews undertaken for accepted manuscripts. Reviewers are asked to complete a checklist review form and are provided with free text boxes to elaborate on their assessment. These free text comments are reproduced below.

## ARTICLE DETAILS

### Title (Provisional)

Development of actionable quality indicators and an implementation toolkit for perioperative opioid stewardship in colorectal cancer in the UK Yorkshire and Humber region: A modified-RAND consensus study.

### Authors

Alderson, Sarah; Thomas, Caroline; Rossington, Hannah; Connearn, Emily; Howell, Simon

---

## VERSION 1 - REVIEW

---

| | |
|---|---|
| **Reviewer** | **1** |
| **Name** | **Bicket, Mark C.** |
| **Affiliation** | **University of Michigan, Anesthesiology and Health Management and Policy** |
| **Date** | **06-May-2025** |
| **COI** | **None** |

---

Thank you for the opportunity to review this original research manuscript for BMJ Open on developing actionable quality indicators and an associated toolkit for bowel cancer opioid stewardship.

Please ensure reporting follows suggested guidelines for consensus studies such as 10.1371/journal.pmed.1004326

Title: Consider “colorectal”, “colon and rectum”, or more anatomically specific alternatives to ‘bowel’

Abstract background: Readers may have some confusion given the reference to ‘post-operative chemotherapy’ as one example of a potential “opioid-related complication”. Please revise or clarify.

Abstract background: Readers would benefit from clarifying the group that “those” refers to

Abstract methods: Please include details relevant to the consensus process, including dates (or year), some sample of backgrounds involved for “experts”

Abstract results: Please specify the group or entity who should demonstrate presence of the protocol. Also, are post-operative pain assessments relevant to inpatient care, outpatient care, or both?

Abstract results: Please clarify what the range of indicator barriers per indicator means and the context for ERIC strategies.

The abstract conclusions mention key stakeholders, though an opportunity exists to clarify who they are besides the “public” “patients” and “experts”

The strengths and limitations reads well, and would benefit from additional specification of the topic of focus of opioid stewardship among colorectal cancer patients.

Introduction - please detail the key or important stakeholders relevant to the quality of care for colorectal surgical patients.

Methods - Please include a statement regarding ethics board approval or exemption

Methods - page 7 lines 25-32 - Please describe the backgrounds of the invited panelists and their potential relevance to the full set of relevant stakeholders for opioid stewardship for colorectal cancer surgical patients

Methods - Specify where appropriate the number of experts, patients, stakeholders, and others involved in this study. How many of the PPG panel members joined the effort (response rate)?

Methods- Please describe when the various meetings or parts of the consensus process took place.

Methods - Please include samples of the surveys for the various meetings as supplementary material.

Methods - Page 8 line 40 - Please clarify who “we’ is and in what settings this discussion took place

Methods - Clarify what is YCR BCIP?

The list of 73 indicators is likely appropriate for supplementary materials. Please consider an abbreviated table with the most important elements based on scoring or another measure. Also, why were at least two of the items not discussed?

Results stage 1 - it is unclear how the literature review informed the expert panel and teh step between the 110 potential indicators that were extracted and the expert panel suggested 83 of those indicators. What steps happened in between or what decision making took place?

The composition of the expert panel mentioned in the results is unclear. What backgrounds / experiences did these “experts” have?

Table 2 - the relevant groups to involve in the stewardship protocol remain unclearly defined

Discussion - Please comment on the types of indicators, given “process” and ‘outcome” indicators were described in the introduction. Why might outcome indicators not be prioritized by the expert panel?

Discussion - the concept of “strong” vs. “weak” opioids improperly gives the view that some opioids are without risk, or with low enough risk to not warrant as careful attention as others. A strong body of evidence supports the concept that all opioids confer risk and that risk is dose dependent. Either modify the statements to avoid the use of strong and weak, which enhance the generalizability of the work, or describe this as a notable limitation to other surgical environments given it is clear that all opioids confer risk of overdose and that risk is dose dependent.

---

## VERSION 1 - AUTHOR RESPONSE

*We would like to thank the reviewer for the comprehensive review and have detailed the changes in the article we have made to each point in response to the helpful feedback.*

Please ensure reporting follows suggested guidelines for consensus studies such as 10.1371/journal.pmed.1004326

*We have added the ACCORD checklist to the supplementary material (S1).*

Title: Consider “colorectal”, “colon and rectum”, or more anatomically specific alternatives to ‘bowel’

*We have changed ‘bowel’ to ‘colorectal’ in the title and throughout the article.*

Abstract background: Readers may have some confusion given the reference to ‘post-operative chemotherapy’ as one example of a potential “opioid-related complication”. Please revise or clarify.

*We have made this clearer that adjuvant chemotherapy might be delayed:*

“high risk of adverse outcomes from opioid-related complications, including delayed discharge and adjuvant chemotherapy,”

Abstract background: Readers would benefit from clarifying the group that “those” refers to

*We have changed 'those' to "patients undergoing colorectal cancer surgery"*

Abstract methods: Please include details relevant to the consensus process, including dates (or year), some sample of backgrounds involved for "experts"

*We have added information on year and that the experts are national peri-operative opioid stewardship experts.*

Abstract results: Please specify the group or entity who should demonstrate presence of the protocol. Also, are post-operative pain assessments relevant to inpatient care, outpatient care, or both?

*We have added 'Trust' presence of an opioid stewardship protocol, and the pain assessments are recommended for inpatients.*

Abstract results: Please clarify what the range of indicator barriers per indicator means and the context for ERIC strategies.
*We have changed this sentence to: The number of barriers identified ranged from 8 to 22 per indicator. 49 different implementation strategies were identified for the toolkit (range 32 to 45 per indicator).*

The abstract conclusions mention key stakeholders, though an opportunity exists to clarify who they are besides the "public" "patients" and "experts"

*We have changed this section to: Our study identified four actionable quality indicators and an implementation toolkit that represents consensus among UK regional anaesthetists, peri-operative opioid stewardship experts and patients in defining quality of care in opioid stewardship for CRC surgery.*

The strengths and limitations reads well, and would benefit from additional specification of the topic of focus of opioid stewardship among colorectal cancer patients.

*Thank you, we have added this to the strengths and limitations section.*

Introduction - please detail the key or important stakeholders relevant to the quality of care for colorectal surgical patients.

*We have added in: Indicators should be easy to understand by key interested parties such as the multi-disciplinary CRC team, managers, commissioners and patients*

Methods - Please include a statement regarding ethics board approval or exemption

*We have added a statement regarding this, although the details are removed as per BMJ Open review guidelines*.

Methods - page 7 lines 25-32 - Please describe the backgrounds of the invited panelists and their potential relevance to the full set of relevant stakeholders for opioid stewardship for colorectal cancer surgical patients

*We have amended this to: We invited panelists based on their (inter)national involvement in guideline development regarding opioid use in (colorectal) surgery for their expertise (n=2) and key local leaders for quality improvement in CRC care (MDT lead anaesthetists and surgeons) for their real-world knowledge of barriers to, and implementation of, potential quality indicators (n=31) from 14 National Health Service trusts in the [XXX] region (population 5.4 million).*

Methods - Specify where appropriate the number of experts, patients, stakeholders, and others involved in this study. How many of the PPG panel members joined the effort (response rate)?

*We have added this to the methods as per above*.

Methods- Please describe when the various meetings or parts of the consensus process took place.

*We have clarified that the PPG meeting was online and added a placeholder for the final meeting (for anonymisation).*

Methods - Please include samples of the surveys for the various meetings as supplementary material.

*These have now been added for rounds 2-4 to the supplemental materials*.

Methods - Page 8 line 40 - Please clarify who “we’ is and in what settings this discussion took place

*We have clarified that this is the research team.*

Methods - Clarify what is YCR BCIP?

*Apologies, this is one of the project identifiers removed from the above text. We have now removed it here for consistency in the review process.*

The list of 73 indicators is likely appropriate for supplementary materials. Please consider an abbreviated table with the most important elements based on scoring or another measure. Also, why were at least two of the items not discussed?

*We have moved this table to supplements (Supplementary table 1) and kept the scores for the four indicators that were accepted only in the results section. The four indicators not discussed were excluded from Round 4 due to being rated 'not actionable'. We have added this information under Round 4 results.*

Results stage 1 - it is unclear how the literature review informed the expert panel and the step between the 110 potential indicators that were extracted and the expert panel suggested 83 of those indicators. What steps happened in between or what decision making took place?

*We have added further information to this stage to make this clearer: The methods and results of the literature search have been described elsewhere (24). The search identified 558 papers, of which 83 papers had full-text review. 30 papers met inclusion criteria and had their references reviewed, leading to review of a further 35 full-text papers, of which 17 additional papers were included. In total, 128 potential quality indicators were extracted from the 47 included papers. Twelve relevant guidelines were identified with 110 potential indicators extracted. The expert panel additionally suggested 83 potential indicators. All 420 potential indicators from the three sources were reviewed for duplication by the research team, grouped and rephrased where necessary to give 73 potential indicators for the online survey. The full list of 73 indicators and the scores at each following stage are given in Supplementary table 1.*

The composition of the expert panel mentioned in the results is unclear. What backgrounds / experiences did these "experts" have?

*We have added further description to the participants in the panel: Ten participants formed the expert panel and responded to the online survey. These included nine colorectal cancer MDT anaesthetic leads from eight trusts within the [XXX] region and one national expert on peri-operative opioid use.*

Table 2 - the relevant groups to involve in the stewardship protocol remain unclearly defined

*We have amended this to: Presence of a Trust perioperative opioid stewardship protocol for colorectal cancer surgery. We have also added to the definition of this indicator: Presence of an opioid stewardship protocol to reduce perioperative opioid use in colorectal cancer surgery, covering the patient journey through pre-operative assessment, operative, recovery and postoperative inpatient care, discharge and follow up of patients, including use of multimodal analgesia, adjuncts such as NSAID's and medications that may limit pain experience such as antiemetics for prescribing clinicians and care staff.*

Discussion - Please comment on the types of indicators, given “process” and ‘outcome” indicators were described in the introduction. Why might outcome indicators not be prioritized by the expert panel?

*We have added the following to explain why process indicators were preferred over outcome indicators: Process indicators were preferred over patient outcome indicators as they are more feasible to measure and for the hospital teams to action. Outcome indicators such as indicator 63 (Patient still taking opioids at 90 days to be reviewed) were not feasible to measure within existing EHRs that do not link primary and secondary care data or the time frame to outcome was considered too long.*

Discussion - the concept of “strong” vs. “weak” opioids improperly gives the view that some opioids are without risk, or with low enough risk to not warrant as careful attention as others. A strong body of evidence supports the concept that all opioids confer risk and that risk is dose dependent. Either modify the statements to avoid the use of strong and weak, which enhance the generalizability of the work, or describe this as a notable limitation to other surgical environments given it is clear that all opioids confer risk of overdose and that risk is dose dependent.

*We have modified this to: Senior clinician review of ‘strong’ opioids on discharge (as defined in the British National Formulary).*

*We have also further explained this further up in the discussion: would not be achievable in a resource-limited healthcare system despite all opioids having risks of adverse effects (indicator 53 – senior review of need for discharge opioid medication)*

*We agree with the reviewer that all opioids have risks and the dose dependency of these. Within the UK, the British National Formulary (BNF) is the national formulary of the NHS and details medicines commonly prescribed in the UK, along with their uses, cautions, contraindications, side-effects, dosage and relative costs. It separates opioids into those it considers ‘strong’ and ‘weak’. We have therefore included this definition as above, which was also used throughout the study.*
